# BECC438b TLR4 agonist supports unique immune response profiles from nasal and muscular DTaP pertussis vaccines in murine challenge models

**DOI:** 10.1128/iai.00223-23

**Published:** 2024-02-07

**Authors:** Megan A. DeJong, M. Allison Wolf, Graham J. Bitzer, Jesse M. Hall, Nicholas A. Fitzgerald, Gage M. Pyles, Annalisa B. Huckaby, Jonathan E. Petty, Katherine Lee, Mariette Barbier, Justin R. Bevere, Robert K. Ernst, F. Heath Damron

**Affiliations:** 1 Department of Microbiology, Immunology, and Cell Biology, West Virginia University, Morgantown, West Virginia, USA; 2 Vaccine Development Center at West Virginia University Health Sciences Center, Morgantown, West Virginia, USA; 3 Department of Microbial Pathogenesis, University of Maryland School of Dentistry, Baltimore, Maryland, USA; Stanford University, Stanford, California, USA

**Keywords:** *Bordetella pertussis*, whooping cough, pertussis, DTaP, pertussis toxin, pertussis toxoid, FHA, vaccines, leukocytosis, bacterial challenge, pertussis mouse model, vaccine evaluation, BECC, lipid A, adjuvant, TLR4 agonist

## Abstract

The protection afforded by acellular pertussis vaccines wanes over time, and there is a need to develop improved vaccine formulations. Options to improve the vaccines involve the utilization of different adjuvants and administration via different routes. While intramuscular (IM) vaccination provides a robust systemic immune response, intranasal (IN) vaccination theoretically induces a localized immune response within the nasal cavity. In the case of a *Bordetella pertussis* infection, IN vaccination results in an immune response that is similar to natural infection, which provides the longest duration of protection. Current acellular formulations utilize an alum adjuvant, and antibody levels wane over time. To overcome the current limitations with the acellular vaccine, we incorporated a novel TLR4 agonist, BECC438b, into both IM and IN acellular formulations to determine its ability to protect against infection in a murine airway challenge model. Following immunization and challenge, we observed that DTaP + BECC438b reduced bacterial burden within the lung and trachea for both administration routes when compared with mock-vaccinated and challenged (MVC) mice. Interestingly, IN administration of DTaP + BECC438b induced a Th1-polarized immune response, while IM vaccination polarized toward a Th2 immune response. RNA sequencing analysis of the lung demonstrated that DTaP + BECC438b activates biological pathways similar to natural infection. Additionally, IN administration of DTaP + BECC438b activated the expression of genes involved in a multitude of pathways associated with the immune system. Overall, these data suggest that BECC438b adjuvant and the IN vaccination route can impact efficacy and responses of pertussis vaccines in pre-clinical mouse models.

## INTRODUCTION

While pertussis, colloquially known as whooping cough, was nearly eradicated in the United States in the 1950s, several cyclic increases in the number of cases over the past 20 years have been observed (1, 2). This resurgence can be correlated to the use of acellular pertussis (aP) vaccines. aP vaccines (DTaP/Tdap) have several distinctions from whole-cell pertussis vaccines (DTP/wP) including (i) the induction of a Th2 dominant immune response due to the alum adjuvant (3, 4), (ii) the loss of the pertactin (PRN) antigen from many strains of *Bordetella pertussis* (5, 6), and (iii) waning immunity with asymptomatic infections that contribute to transmission. Unlike the aP vaccine, wP formulations offer long-lasting protection via the induction of a Th1-polarized immune response and the inclusion of the entire *B. pertussis* bacterium, which presents more antigens against which the immune system can mount an immune response (4, 7, 8). However, the robust immune response elicited by the wP vaccine comes at the cost of occasional deleterious side effects, which has prompted vaccine hesitancy and lawsuits (9–11). To avoid these concerns, a protein subunit acellular vaccine was developed to replace wP in the United States (12, 13). Initially, the data from aP vaccine studies demonstrated that the acellular formulation induced antibodies to *B. pertussis* antigens at similar levels to what was seen from the wP formulation (13–15). However, as time went on, the issue of waning vaccine immunity became apparent with the number of pertussis cases beginning to rise in the years after the implementation of the aP formulation (12, 16). One consequence of waning immunity is the issue of asymptomatic nasal carriage, which occurs when an individual harbors the bacteria within their nasal mucosa but does not show signs of infection (17, 18). The pathogen can then be passed on to highly susceptible populations, specifically those less than 1 year of age, resulting in illness, hospitalization, and even death (2). As a result, there is an ongoing effort in the field to improve aP vaccine formulations such that their resulting immune responses are more similar to a natural infection, as a natural infection provides the longest known duration of protection against *B. pertussis* (7, 19, 20). There are many ways in which pertussis vaccines could be improved: different routes of vaccine administration could be utilized (20–24), more immunogenic antigens could be implemented (25–27), and improved adjuvants could be incorporated to either replace or enhance the response to alum (28–30).

Currently, pertussis vaccines are only administered intramuscularly (IM). IM vaccines deliver the antigens to the draining lymph nodes. From there, the antigens are presented to immune cells, which allows for the induction of a systemic immune response and the production of antibodies that will bind the pathogen upon re-encounter (31). While IM vaccination offers protection from disease, it does not completely address the issue of nasal carriage, that is, upon exposure to *B. pertussis*, vaccinated hosts can still be colonized by the bacterium in their nasal cavity (32). Additionally, studies have shown the value of tissue-resident memory T cell responses, which may not be gained through IM vaccination, and their importance in clearance and protection (18, 33). One alternative route of vaccine administration that has shown to be promising in regard to controlling pertussis infections is the intranasal (IN) route of vaccination (30, 34). With IN vaccination, the antigens are also delivered to the draining lymph nodes, which results in a systemic immune response and the induction of antibodies. However, there is also the activation of immune cells in the nasal mucosa, which results in the induction of a mucosal immune response (35). As a result, there is the production of antigen-specific IgA antibodies, as well as the localization of immune cells to the site of colonization. Multiple studies have demonstrated the effectiveness of IN vaccines in protecting both animal models and people against *B. pertussis* infections (24, 30, 34, 36, 37).

aP vaccines utilize the adjuvant alum, to which the antigens included in the formulation are adsorbed, allowing for their presentation to immune cells (38). Alum elicits a Th2-polarized immune response, which leads to the downstream activation of humoral immunity and production of antibodies (39, 40). Conversely, both the wP vaccine and natural infection activate a Th1-polarized immune response, which induces more inflammation and is characterized by the activation of cell-mediated immunity (7, 41). The current research supports the importance of cell-mediated immunity in the clearance of *B. pertussis* (7). To ameliorate this issue, a number of different Th1-polarizing adjuvants could be utilized in the aP vaccine, such as beta glucans, TLR9 ligands, and TLR4 agonists (28, 30, 42, 43).

One adjuvant category of particular interest is lipid A mimetics, which have been demonstrated to induce a Th1-polarized immune response by binding to Toll-like receptor 4 (TLR4) (43, 44). Lipopolysaccharide (LPS), a membrane component of Gram-negative bacteria, canonically binds to TLR4 and its co-receptor, MD-2 (45, 46). LPS consists of the O antigen, a core, and lipid A, which varies between bacterial species and is responsible for most of the deleterious side effects that stem from a Gram-negative bacterial infection (46, 47). The structure of lipid A determines the downstream immune response; therefore, in the case of vaccination, the ability to generate novel lipid A structures that activate specific inflammatory pathways would make for powerful adjuvants (43, 48, 49). It has been appreciated that the potent immune response of the wP formulation results from both the number of antigens available and the immunostimulatory effect of *B. pertussis*’ lipooligosaccharide (LOS) molecule (50, 51). We hypothesize that the use of a vaccine formula containing an engineered lipid A mimetic could confer the ideal aspects of both types of pertussis vaccines: the protective immunity of wP and the safety of aP. For these studies, we utilized a novel lipid A structure TLR4 agonist (BECC438b) that was engineered using Bacterial Enzymatic Combinatorial Chemistry (BECC) technology (52). An attenuated strain of *Yersinia pestis* (*Yp*) is grown at different temperatures—either 26°C or 37°C—resulting in the synthesis of variable lipid A structures (42, 49, 52, 53). The end result is a breadth of lipid A molecules which have been shown to induce either a highly biased Th1 response, a Th2 response, or a balanced Th1/Th2 response (54). BECC438b is a bisphosphorylated lipid A molecule derived from a *Yp* strain that lacks the C_12:0_ acyltransferase (MsbB) but expresses a functional C_16:0_ acyltransferase (PagP), resulting in a molecule that elicits a more balanced Th1/Th2 immune response when compared with standard lipid A structures (49, 53). BECC has been used to create adjuvants that have been efficacious in pre-clinical vaccines for a number of pathogens including *Yp*, SARS-CoV-2, *Pseudomonas aeruginosa*, and influenza type A (42, 53, 55–57).

Here, we detail the effects of adding the BECC438b adjuvant to the current DTaP formulation, which contains alum. Furthermore, the efficacy of the DTaP + BECC438b formulation administered as an IM or IN vaccine was evaluated. Female CD-1 mice were vaccinated and challenged in order to determine protection via the enumeration of colony-forming units (CFUs) and the quantification of antibodies against various pertussis vaccine antigens. Additionally, lung histopathological analysis was performed with quantitation of cytokines within the lung and trachea to determine the extent of inflammation resulting from vaccination and bacterial challenge. Finally, RNA sequencing (RNAseq) analysis was performed to further differentiate the downstream immune processes that resulted from vaccination with BECC438b and subsequent *B. pertussis* challenge (Fig. S1). Overall, the addition of BECC438b to DTaP administered IM was able to improve serological immune responses as well as reduce bacterial burden within the lower respiratory tract. Furthermore, when DTaP + BECC438b was administered IN, there was an increase in the downstream activation of immune response pathways (such as those involved in both activation and regulation of the immune system) as well as an increase in IgA production, an indicator of mucosal immunity.

## MATERIALS AND METHODS

### Vaccine and adjuvant composition

The GSK Infanrix formulation of DTaP was used and diluted to 1/40th the human dose (12.5 µL vaccine diluted to 50 µL total volume administered in 0.9% saline) and was administered no longer than 1 h after preparation. BECC438b was reconstituted using endotoxin-free water. After reconstitution, BECC438b (50 µg per dose) was combined with Infanrix DTaP, and the mixture was turned end-over-end for 2 h. All vaccines administered to mice contained the alum adjuvant at the dose included in the base Infanrix formulation.

### Vaccine administration

Female CD-1 (outbred; strain code 022) mice aged 4 to 5 weeks were obtained from Charles River Laboratories. Mice were administered 50 µL of vaccine or vehicle control IM in the right thigh. For intranasal vaccines, mice were anesthetized with ketamine/xylazine (77 mg/kg of body weight of ketamine and 7.7 mg/kg of xylazine) and 25 µL of the vaccine was administered into each nostril. Mice were boosted with the same vaccine formulations 21 days after priming. All murine infection experiments were performed according to protocols approved by the West Virginia University Animal Care and Use Committee (protocol numbers: 1602000797 and 1602000797_R1).

### *B. pertussis* strains and growth conditions

*B. pertussis* strain UT25Sm1 was used for murine challenge. The *B. pertussis* strain has been fully genome sequenced (UT25Sm1 NCBI Reference Sequence: NZ_CP015771.1). UT25 was originally isolated in Texas in 1977 (58). UT25Sm1 was cultured on Bordet Gengou (BG) agar (Difco) supplemented with 15% defibrinated sheep’s blood (Hemostat Laboratories) and streptomycin 100 µg/mL. *B. pertussis* was incubated at 36°C for 48 h and then transferred to Stainer-Scholte liquid medium (SSM). SSM liquid cultures were incubated for 24 h at 36°C, with shaking at 180 rpm until reaching an optical density at 600 nm (OD_600_) of ~0.6, at which time cultures were diluted for the challenge dose. For challenge with a liquid inoculum *B. pertussis*, the mice were anesthetized with 77 mg/kg of body weight of ketamine and 7.7 mg/kg of xylazine and inoculated with 20 µL (10 µL per nostril) of the challenge dose (2  ×  10^7^ CFU). For mice challenged with aerosolized *B. pertussis*, the mice were placed into a dosing chamber and 20 mL of the challenge dose (10^9^ CFU/mL) was nebulized for 10 min (59).

### Vaccine challenge model

At 35 days post prime, mice were challenged with IN 2 × 10^7^ CFUs of *B. pertussis* (10 µL per nostril or nebulized challenge). At day 3 post-challenge, mice were euthanized, and blood was collected by cardiac puncture. Three days post-challenge was selected as our end-point based on our previous studies (28, 30). No mice were excluded from analysis. A complete blood cell count with differential was performed using a ProCyte Dx hematology analyzer (IDEXX), and serum was separated by centrifugation through a BD Microtainer blood collector and stored at −80°C until analysis. The trachea and lungs were removed and homogenized together. Lungs and trachea were suspended in 2 mL of sterile phosphate buffered saline (PBS) in GentleMACS C tubes (Miltenyi; Cat. Number: 130-096-334) and homogenized using a GentleMACS Octo Dissociator with Heaters (Miltenyi) with the m_lung_02 setting. The lung/trachea homogenates were centrifuged at 14,000 × *g* for 4 mins, and supernatants were stored at −80°C until cytokine and antibody analyses were performed. The nasal lavage was collected by flushing 1 mL of 1× PBS through the nasal cavity and collecting the wash in an Eppendorf tube. A scalpel was used to cut out the hard palate and nasal-associated lymphoid tissue (NALT). The hard palate and NALT were placed in a culture tube with 1 mL of 1× PBS, homogenized with a polytron, and then filtered through a 70-μM filter. To isolate the septum, the nasal lavage was collected, and the hard palate was removed for NALT collection. Then, the skin of the skull was removed, and a cut was made anterior to the eyes. Bacterial burden was determined in the lung/trachea, nasal lavage, NALT, and septum by CFUs using serial dilutions. Serial dilutions were done in PBS and then plated on BG agar containing streptomycin (100 µg/mL) to ensure that only *B. pertussis* isolate UT25Sm1 was cultured.

### Serological analysis of *B. pertussis*-specific antibodies

On the day of mouse processing (euthanasia and dissection), a cardiac puncture was performed on each mouse. Approximately 1 mL of blood was removed from each mouse. The samples were placed into serum separator tubes (Cat. Number: 365967; BD) centrifuged for 2 min at 14,000 × *g*. Serum was collected and stored at −80°C until analyses were performed. Serological responses specific to *B. pertussis* antigens were quantified by enzyme-linked immunosorbent assay (ELISA). High-binding microtiter plates were coated with pertussis toxin (PT) (50 ng/well) (PT#180, LIST Biologicals), filamentous hemagglutinin (FHA) (50 ng/well) (Enzo Life Sciences), or PRN (50 ng/well) (PRN#187, LIST Biologicals) as described in Boehm et al. (34). For serological responses to *B. pertussis*, UT25Sm1 was cultured to an OD_600_ of ~0.6 and diluted down to an OD_600_ of 0.245 and microtiter plates were coated with 50 µL of bacterial solution per well overnight. After coating, the plates were washed with PBS + 0.05% vol/vol Tween 20 (Cat. Number: P1379-1L, Sigma-Aldrich) (PBS-T) and blocked with 5% non-fat dry (NFD) milk in PBS-T overnight at 4°C. Blocked plates were washed with PBS-T, and then, serum (1:50) from vaccinated and challenged mice was prepared in 5% NFD milk in PBS-T. All samples were serially diluted (1:2). After 2 h incubation at 37°C, plates were washed and incubated with goat anti-mouse-IgG alkaline-phosphatase-conjugated antibodies (IgG:1030-04, Southern Biotech) (1:2,000) for 1 h at 37°C. For IgG isotyping, plates were washed and incubated with either goat anti-mouse IgG2a alkaline-phosphatase-conjugated antibody (Southern Biotech, Cat. Number: 1081-04), goat anti-mouse IgG2b alkaline-phosphatase-conjugated antibody (Southern Biotech, Cat. Number: 1091-04), or goat anti-mouse IgG1 (Southern Biotech, Cat. Number: 1071-04) for 1 h at 37°C (all antibodies diluted 1:20,00 in 5% NFD milk in PBS-T). Plates using the alkaline phosphatase-conjugated secondary antibody were then washed and incubated with Pierce *p*-Nitrophenyl Phosphate (PNPP) (Cat. Number: 37620; Thermo Fisher Scientific) following the manufacturer’s instructions. The absorbance of the plates was read at OD_405_ using a Synergy H1 plate reader (BioTek). Positive antibody titers were determined as any values above the baseline as determined by the final dilution at which the values were two times the average of blanks. To determine the proportion of each IgG isotype present in the serum, the end-point titer value for one isotype was divided into the total summation of all end-point titer values across all isotypes measured (28, 30, 60–62). Sera obtained from mice vaccinated with either 1/5th the human dose of wP or DTaP were used in the assay as technical positive controls of the assay.

### Analysis of mucosal IgA antibodies

On the day of processing, the nasal lavage and lung/trachea supernatants were collected. The nasal lavage was stored at −80°C until analysis, and the lung/trachea homogenates were centrifuged at 15,000 *g* × 3 min. Upon completion of centrifugation, the supernatants of the lung and trachea were collected and transferred to new Eppendorf tubes and stored at −80°C until analysis. For analysis of IgA antibodies, UT25Sm1 was cultured to an OD_600_ of ~0.6 and diluted down to an OD_600_ of 0.245 and microtiter plates were coated with 50 µL of bacteria per well and incubated overnight. After coating, the plates were washed with PBS + 0.05% vol/vol Tween 20 (Cat. Number: P1379-1L, Sigma-Aldrich) (PBS-T) and blocked with 5% non-fat dry (NFD) milk in PBS-T overnight at 4°C. Blocked plates were washed with PBS-T, and then, nasal lavage (no initial dilution) or lung supernatant (1:4) samples were prepared in 5% NFD milk in PBS-T. All samples were serially diluted (1:2) down the plate. After 2 h incubation at 37°C, plates were washed and incubated with goat anti-mouse IgA heavy-chain secondary antibody conjugated to horseradish peroxidase (HRP) (Cat. Number: NB7504, Novus Biologicals) (1:4,000) for 1 h at 37°C. The plates that utilized the HRP enzyme were then washed and incubated with TMB substrate (Cat. Number: 421101, BioLegend). The absorbance of the plates was read at OD_605_ using the Synergy H1 plate reader mentioned above. Positive antibody titers were determined as any values above the baseline (set at two times the average of blanks).

### Analysis of cytokines within the lung and trachea supernatants

To quantify inflammatory cytokines at the site of infection, lung/trachea homogenate supernatants were prepared, as suggested by the kit manufacturer, and diluted 1:2. Quantitative analysis of cytokines was performed using a custom kit from R&D Systems which analyzed CXCL-13, IL-1β, IL-4, IL-5, IL-6, IL-10, IL-12p70, IL-17A, TNF-α, and IFN-γ according to the manufacturer’s instructions. Samples were run on a Magpix (Luminex) instrument. Bead counts below 35 were invalidated and not used in the final quantification of cytokine levels.

### Histological analysis of lung tissue

Upon euthanasia, the lungs were removed and the right lobe was placed into 10% formalin for 48 h at 4°C. The right lobe was then embedded in paraffin and stained with H&E (hematoxylin and eosin) by the West Virginia University Department of Pathology. H&E-stained sections were sent to iHisto for blinded analysis and histological scoring by a board-certified pathologist. The H&E-stained slides were reviewed for both acute and chronic lung inflammation present at parenchymal, perivascular, and peribronchial regions. Inflammation and damage to the epithelial cells in bronchi and bronchioles were also evaluated. Semiquantitative scores were made following standard toxicologic scoring criteria (0—none, 1—minimal, 2—mild, 3—moderate, 4—marked, and 5—severe). Individual scores of the evaluated parameters were recorded. The acute inflammation score for acute or chronic inflammation score for chronic inflammation was calculated by adding the scores of individual parameters. The total inflammation score was calculated by adding the acute inflammation score, chronic inflammation score, and the inflammation score for epithelia in bronchi or bronchioles.

### RNAseq analysis of the lung

Transcriptomic analysis was performed as described previously (55). Lungs of mice were excised, and the left lobe of the lung was placed into TRIzol (Cat. Number: 15596026; Thermo Fisher) at a ratio of 1:3 volumes of the sample to TRIzol. RNA was purified using the Direct-zol RNA MiniPrep Kit (Zymo Research; R2053) following the manufacturer’s protocol. RNA quantity was measured with a Qubit 3.0 Fluormeter using the RNA high sensitivity (Cat. Number: Q33216; Life Technologies), and RNA integrity was assessed on an Agilent 4200 TapeStation System. RNA was treated with DNAse (Qiagen), per manufacture’s protocol, before library preparation. Illumina sequencing libraries were prepared with TruSeq Stranded with RiboZero Plus depletion. Resulting libraries passed standard Illumina quality control PCR and were sequenced on an Illumina NovaSeq platform at Admera Health (South Plainfield, NJ). A total of ~200 million 2 × 150-bp reads were acquired per sample. The reads were trimmed for quality and mapped to the *Mus musculus* reference genome using CLC Genomics Version 21.0.5. An exported gene expression browser table is provided at https://data.mendeley.com/. Statistical analysis was performed with the Differential Gene Expression tool, and genes were annotated with the reference mouse Gene Ontology (GO) terms. Genes with a Bonferroni-corrected *P* value of < 0.05 were considered differentially regulated. Genes of interest were plotted in a heat map that was generated in GraphPad Prism version 9.0. Genes that were differentially regulated were further analyzed via the online WEB-based GEne SeT AnaLysis Toolkit using over-representation analysis using the mouse enrichment category Gene Ontology and biological process (63). Heat maps were generated using Morpheus (64). The bubble plot of GO biological processes was created using RStudio and the ggplot2 package as described in the protocol by Bonnot et al. (65). Venn diagrams of common activated genes were created using the VennDiagram package of RStudio. Chord diagrams were created via the circlize package of RStudio.

### Statistical analysis

Only histopathological data were scored and analyzed by a researcher blinded to the experimental groups. Statistical analyses were performed using Prism version 9.0 software (GraphPad) on native, non-log transformed data set values. Comparisons between three or more groups were performed via a one-way analysis of variance (ANOVA) or Kruskal-Wallis test based on the normality of the data. Comparisons between two variables were made utilizing unpaired Student’s *t*-test.

## RESULTS

### Intranasal administration of DTaP confers equal or better protection as intramuscular administration against bacterial burden in the respiratory tract

**Fig 1:**
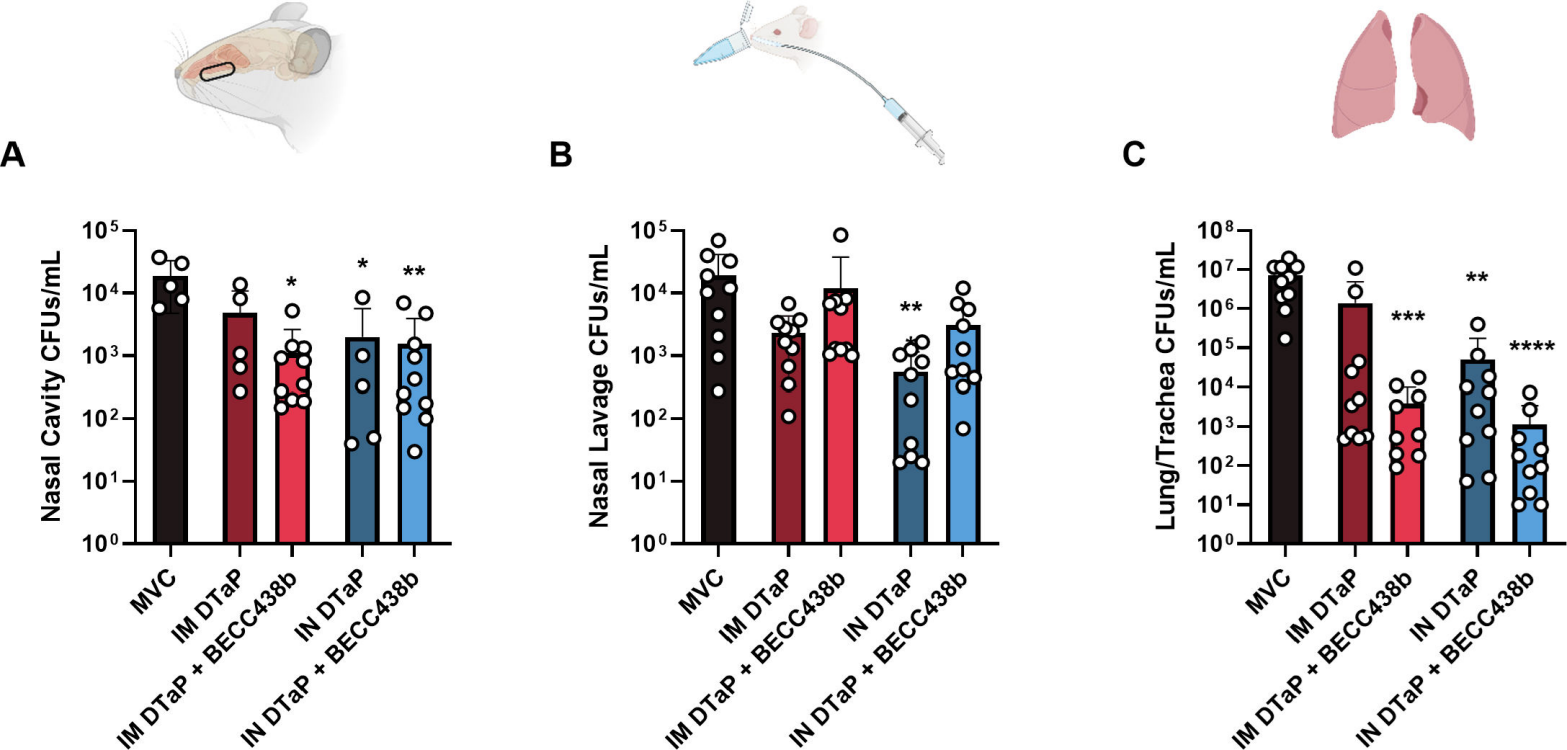
Enumeration of bacterial burden within the respiratory tract of naive or vaccinated mice at 3 days post *B. pertussis* challenge. CFUs were enumerated within the nasal cavity (**A**), nasal lavage (**B**), and the lung/trachea (**C**). Data presented as geometric means ± SD, *n* = 10 per treatment group. Exceptions to this are the IM DTaP- and IN DTaP-vaccinated mice for nasal cavity CFUs (*n* = 5), and no mice were excluded from analysis. Kruskal-Wallis tests were performed to determine statistically significant differences between MVC mice and all other treatment groups. *P < 0.0264, **P < 0.0074, ***P < 0.0004, and ****P < 0.0001. Mann-Whitney tests were performed between each experimental group in order to determine statistically significant differences between each vaccine route and formulation. *P < 0.05. *P*-values that approach statistical significance are indicated. CFU, colony-forming units; MVC, mock-vaccinated, challenged; IM, intramuscular; IN, intranasal.

Previous studies in our lab have characterized the protection of pertussis vaccines in murine models and shown that these vaccines can confer protection in mice when administered both IM and IN (30, 34). Here in this study, we evaluated the effect of the TLR4 agonist BECC438b co-administered as an adjuvant with DTaP by the IM or IN route to evaluate its ability to induce protective immunity against *B. pertussis* challenge (42, 54). To do this, female CD-1 mice were vaccinated with either 1/40th the human dose of DTaP alone or 1/40th DTaP with BECC438b by the IN or IM route. This dose of vaccine was determined based on previous data from our lab which showed that 1/40th human dose in mice is a non-sterilizing dose that confers protection against *B. pertussis* challenge (66). The adjuvant alum was included in the experimental vaccine formulations as it is already adsorbed to DTaP antigens. Two weeks post-vaccination, mice were subjected to challenge with *B. pertussis* isolate UT25Sm1 and euthanized 3 days later to collect tissue samples for analysis. To determine bacterial burden, CFUs were quantified from the lung and trachea, the nasal cavity (including the NALT), and nasal lavage fluid. At 3 days post-challenge, within the nasal tissue, mice vaccinated by the IN route with DTaP or DTaP + BECC438b showed a statistically significant reduction in bacterial burden when compared with the MVC mice (Fig.1A). The addition of BECC438b to the formulation with DTaP did not make a significant difference between the IN groups; however, in the IM groups, only mice vaccinated with DTaP + BECC438b showed significantly lower *B. pertussis* burdens in the nasal tissue (Fig. 1A). Within fluid collected by nasal lavage, IN DTaP again showed reduction in the number of CFUs from bacteria thought to colonize the upper airway external of that bound to the nasal cavity compared with MVC as well as administration IM (*P* = 0.0095) (Fig. 1B). Interestingly, the addition of BECC438b in the vaccine formulation regardless of administration route did not improve nasal lavage CFUs either, in fact, DTaP + BECC438b formulations reduced bacterial burden less than DTaP alone, although these differences were determined to be insignificant (Fig. 1B). Although intranasal administration of respiratory pathogens is a commonly utilized method for modeling pathogen exposure, it utilizes a large dose of bacteria that is then inhaled and colonizes the lungs. With this in mind, we additionally sought to quantify the bacterial burden of the lung and trachea after vaccination and challenge. IN vaccination but not IM with DTaP significantly reduced *B. pertussis* colonization of the lung (Fig. 1C). Here, the addition of BECC438b to DTaP improved bacterial clearance by the IM route (*P* = 0.0892*)*, decreasing CFUs by 352.7-fold. IN vaccination with BECC438b was also lower than IN DTaP alone, reduced significantly by 44.3-fold (*P* = 0.0281*).* Together, these data support the potential efficacy of both the IN route of administration for DTaP which reduced bacterial burden in the nasal tissues and lung post-challenge, and co-formulation of DTaP with adjuvant BECC438b significantly reduced bacterial burden in the lungs of mice vaccinated IN.

### Aerosol challenge with *B. pertussis* provides an alternative model for evaluating vaccines

Preclinical models of pertussis utilize multiple methods of administering bacteria to mice to simulate authentic pathogen exposure. Direct administration of a high dose of the bacterium to the nares is one method of ensuring bacterial transfer into the airway; however, this leads to disease phenotypes that may not recapitulate disease in humans which occurs from a much smaller aerosolized exposure dose. While mice are infected via this method, the large deposition of bacteria may allow colonization past the limits of what experimental vaccines can reduce. The aerosol challenge model of *B. pertussis*, as an alternative, utilizes an aerosolization chamber and nebulizes the dose of bacteria so that it is inhaled and more closely mimics natural exposure. Our lab, in developing this protocol, has observed lower bacterial burden in the lung, nasal lavage, and trachea of naïve mice after aerosol challenge than those challenged by liquid inoculum (59, 67). To build upon the results of Fig. 1 and rectify the potential caveats of the IN administration model, we designed follow-up studies in which mice were vaccinated and then challenged with aerosolized *B. pertussis*. Two weeks following vaccination, mice were placed into a chamber and were exposed to an aerosolized dose of *B. pertussis*. Mice were then euthanized 3 days post-challenge. In the nasal cavity of aerosol-exposed mice, all vaccines conferred a reduction in bacterial burden when compared with MVC mice; however, only IN DTaP vaccination without BECC438b was able to confer a significant limitation (*P* = 0.0121) (Fig. S2A). Within the nasal septum and nasal lavage fluid, no vaccine was observed to significantly impact bacterial burden (Fig. S2B and C). Within the lung and trachea of vaccinated mice, IN vaccination with either formulation significantly reduced bacterial burden, limiting CFUs in a number of samples to the lower limit of detection in the CFU assay (Fig. S2D). While the aerosolization model did not demonstrate any differential effects from IN and IM vaccines with or without BECC428b on bacterial burden in the upper airway, the limited data do suggest that IN DTaP and IN DTaP + BECC428b can prevent bacterial burden in the lung as effectively as the vaccines administered IM.

**Fig 2:**
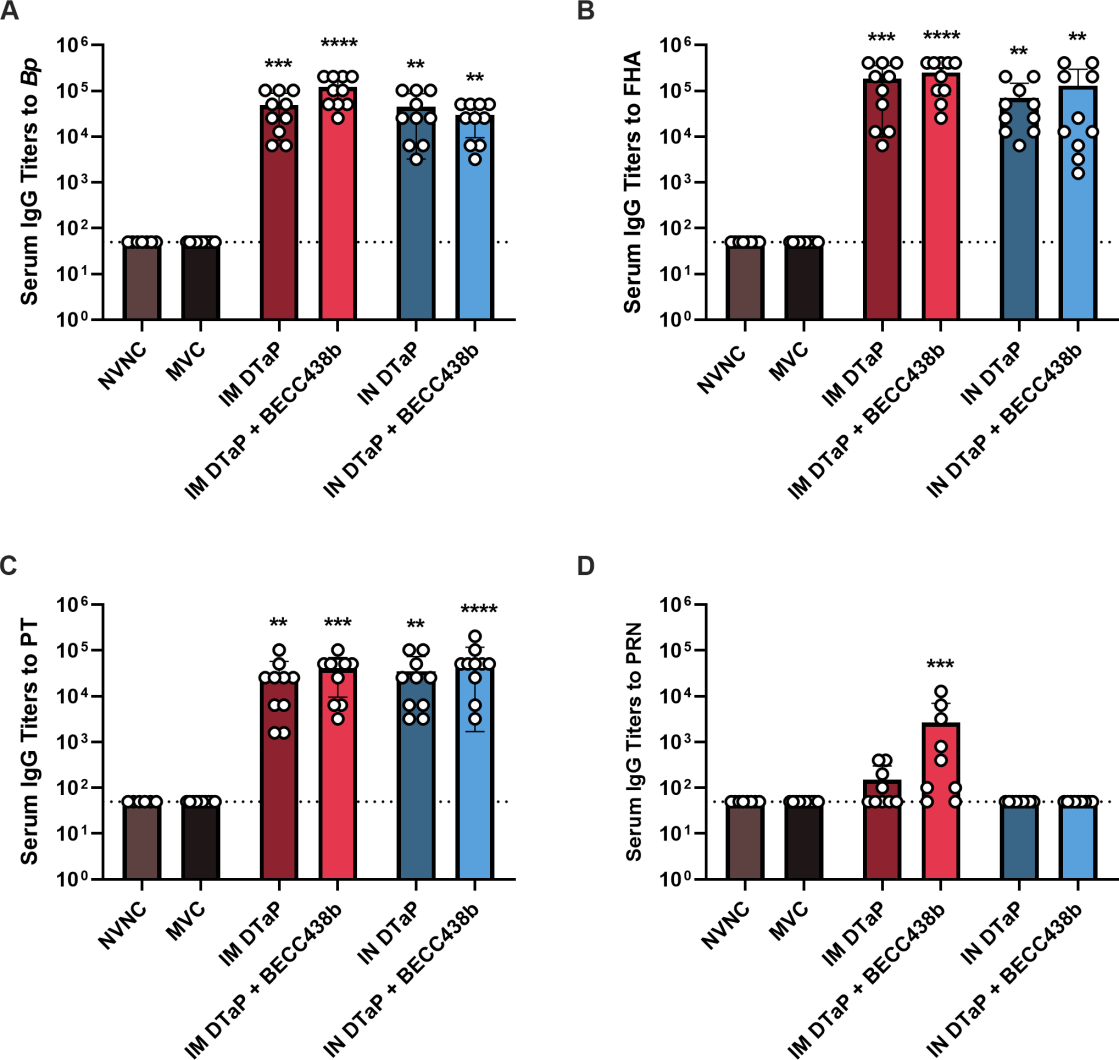
Quantification of serum IgG antibody titers specific to the *B. pertussis* bacterium and pertussis vaccine antigens at 3 days post challenge. IgG antibody titers against the whole *B. pertussis* bacterium (*Bp*) (**A**), FHA (**B**), PT (**C**), and PRN (**D**) were determined by ELISA. Data resented as geometric means ± SD, *n* = 10 per treatment group. Kruskal-Wallis tests were performed to determine statistically significant differences in MVC mice and all other treatment groups. **P < 0.0084, ***P < 0.0009, and ****P < 0.0001. Mann-Whitney tests were performed between each experimental group in order to determine statistically significant differences between each vaccine route and formulation. *P*-values that approach statistical significance (*P* < 0.05) are indicated. MVC, mock-vaccinated, challenged; NVNC, non-vaccinated, non-challenged; IM, intramuscular; IN, intranasal.

### Intramuscular and intranasal administration of DTaP + BECC438b can elicit increased serological responses to *B. pertussis* antigens

Existing aP pertussis vaccines (DTaP and Tdap) are adjuvanted with alum which is known to induce a Th2-dominant humoral immune response (7, 9, 66–68). In *B. pertussis* infections, the functionality of antibodies is paramount for preventing adhesion of the bacterium, upon infection, to the respiratory epithelium in addition to neutralizing toxins (69–72). Numerous adjuvants have been evaluated for their ability to improve immune responses to the pertussis antigens included in the current aP formulations (25, 26, 33, 73, 74). The production of antibodies to the antigens included in DTaP, such as the adhesion proteins FHA and PRN, inhibits the adherence of the bacterium to the respiratory epithelium (72) while others neutralize pertussis toxin. PT is considered an essential virulence factor for *B. pertussis*, resulting in leukocytosis and disruption of vasculature integrity (75–80). Therefore, we sought to determine the extent to which BECC438b in vaccine formulations improved the serological responses against *B. pertussis* infection. Serum IgG antibodies against the whole *B. pertussis* bacterium, in addition to the antigens PT, FHA, and PRN, were quantified by ELISA Fig. S3). In blood samples collected from mice at euthanasia, 3 weeks post-challenge, serum titers of IgG specific to whole *B. pertussis* were elevated across vaccine groups (Fig. 2A). The inclusion of BECC438b in IM administered formulations significantly increased antibody titers (*P* = 0.0272) by threefold, demonstrating a benefit from the adjuvant in generating systemic antibody responses. BECC438b made no significant impact on IN delivered formulations. Anti-FHA antibodies were not statistically different between IM or IN administered DTaP groups; however, IM DTaP + BECC438b generated significantly higher IgG titers than IN DTaP + BECC438b (*P* = 0.0488) (Fig. 2B). All vaccines elicited high anti-PT antibody levels when compared with MVC mice (Fig. 2C). While not significant, the addition of BECC438b to DTaP did result in a slight increase in titer levels for both IM and IN administered vaccines, with a 0.47- and 0.70-fold increase, respectively (Fig. 2C). Despite the small induction of PRN-specific antibodies, we observed a statistically significant increase in titer levels with IM administration of the DTaP + BECC438b formulation when compared to MVC mice (Fig. 2D). Overall, these data suggest that the administration of DTaP by either route induces comparable humoral antibody responses against *B. pertussis* and demonstrates that the combination of DTaP with BECC438b can improve components of these responses in the mouse model.

**Fig 3:**
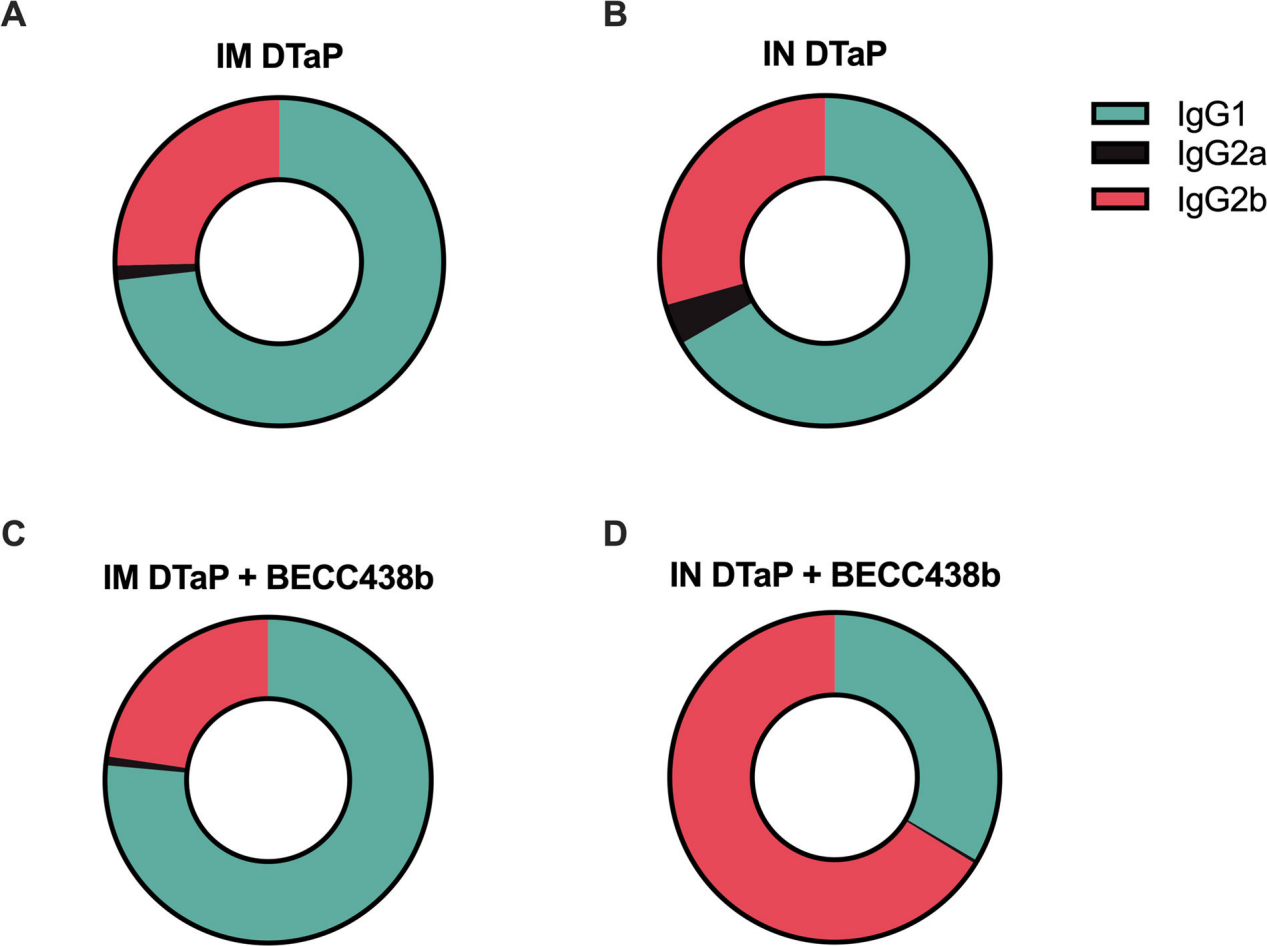
Analysis of IgG isotypes reveals nasal immunization, and BECC438b adjuvant induces Th1 immune responses. A visual part-of-the-whole analysis was created for each experimental group. The proportion of IgG1:IgG2a:IgG2b is shown. The part of a whole graphs visualizes each antibody isotype end-point titer (numerator) and divides that into the total of all antibody end-point titers (denominator). IgG isotype proportions are shown for IM DTaP (**A**), IM DTaP + BECC438b (**B**), IN DTaP (**C**), and IN DTaP + BECC438b (**D**).

### Intranasal administration of DTaP + BECC438b is able to induce a Th1-polarized humoral immune response

One reason underlying the switch from the wP to the aP formulation lies in the strong adverse immunostimulatory effects that can result from wP vaccines (13). While aP formulations decreased the occurrence of post-vaccination side effects, they brought an additional challenge for researchers to address: waning immunity (81). Similar to a natural pertussis infection, wP vaccination elicits a Th1-polarized immune response, with CD4^+^ T cells playing a major role in the clearance of *B. pertussis* from the respiratory tract (41). In contrast, aP vaccines induce strongly Th2-skewed immune responses with the resulting antibodies able to prevent severe disease but lack the ability to confer sterilizing immunity, resulting in transmission in mild or asymptomatic cases (82). Our objective was to determine the T helper cell polarization that can result from the inclusion of BECC438b into both IM and IN DTaP formulations. Using antibody isotype analysis, pre-challenge sera from mice were analyzed for the presence of IgG isotypes: IgG1 (indicative of a Th2-polarized response) and IgG2a/IgG2b (indicative of a Th1-polarized response) specific to the whole bacterium (Fig. S6). Both IM administered vaccines triggered similar immune responses, with 73% of the total IgG isotypes elicited from DTaP being IgG1 and DTaP + BECC438b eliciting IgG isotypes that were 76% IgG1 (Fig. 3A and C). From IN vaccination with DTaP alone, the profile of IgG isotypes was similar to what was seen from IM, with IgG1 making up 67% of the IgG antibodies (Fig. 3B). Interestingly, the addition of BECC438b to IN DTaP changes the proportion of IgG1 antibodies to around 33% of the total IgG and the proportion of IgG2 antibodies to around 67% (Fig. 3D). This suggests that BECC438b likely induces increased Th1 cell-mediated immune responses during IN vaccination, which are highly desirable for generating durable immunity from vaccines.

### The addition of BECC438b to DTaP increases total inflammation post-challenge in the lungs for both IM and IN immunization

**Fig 4:**
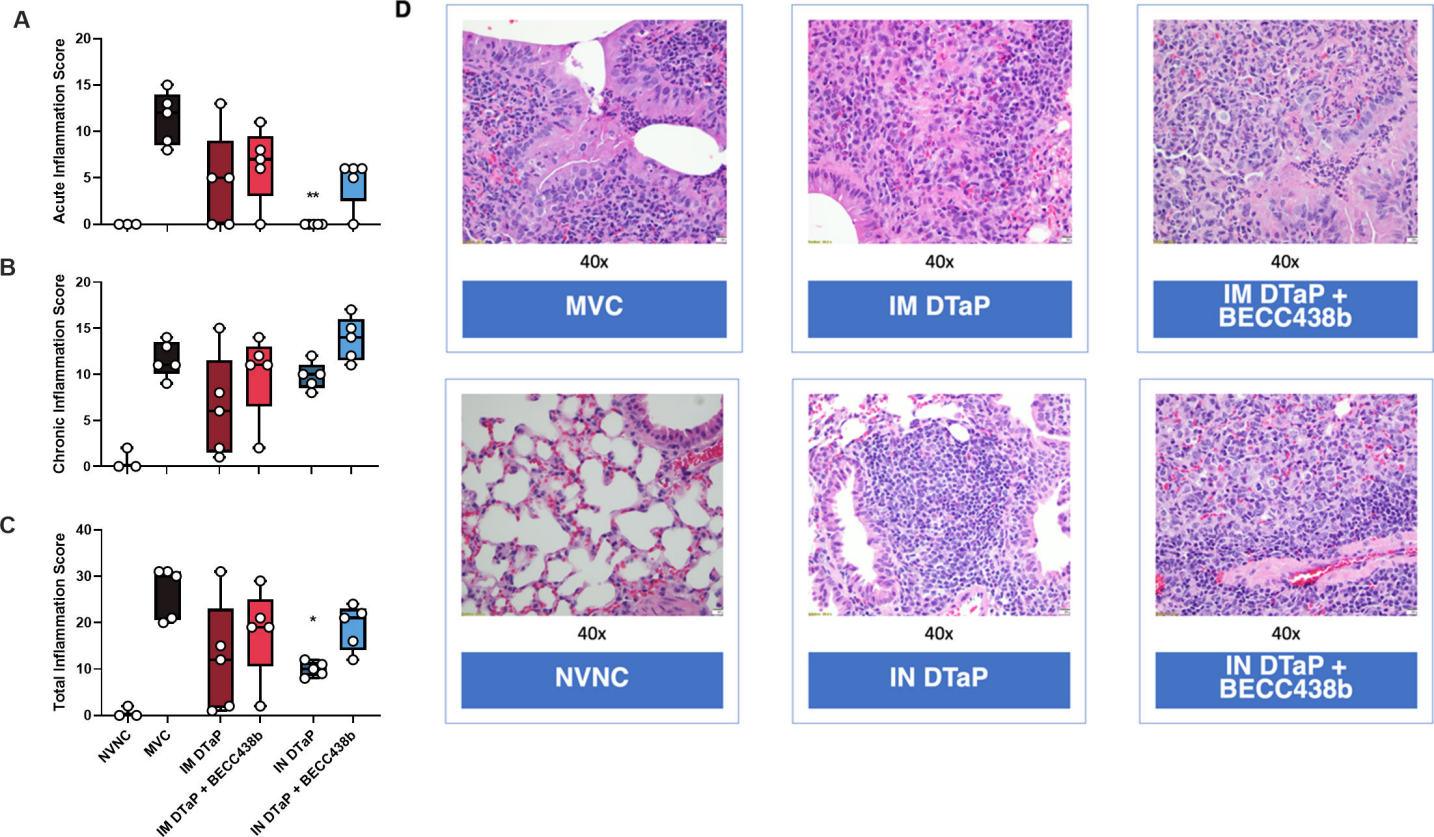
Histopathological analysis of mouse lungs at 3 days post *B. pertussis* challenge. Acute (**A**), chronic (**B**), and total (**C**) inflammation scores were determined for sections of lung tissue from vaccinated and *B. pertussis-*challenged mice by a board-certified pathologist. Representative images of the lung for each experimental group are provided in D. Data presented as geometric means ± SD, *n* = 5 per treatment group. Kruskal-Wallis tests were performed to determine statistically significant differences in MVC mice and all other treatment groups. *P = 0.0311 and **P = 0.0012. Student’s *t*-test was performed between each experimental group in order to determine statistically significant differences between each vaccine route and formulation. *P < 0.05 and **P < 0.01. *P*-values that approach statistical significance are indicated. MVC, mock-vaccinated, challenged; NVNC, non-vaccinated, not-challenged; IM, intramuscular; IN, intranasal.

BECC adjuvants are TLR4 agonists (52). As such, it is expected that some degree of inflammation will occur from their use. Specifically, BECC438b is known to induce a balanced Th1/Th2 immune response (42). To determine the extent of inflammation caused by vaccination with the BECC438b adjuvant, lungs collected from vaccinated mice after *B. pertussis* challenge were fixed and paraffin embedded to allow for H&E staining. A board-certified pathologist blinded to the group designations evaluated the extent of acute, chronic, and total inflammation within the parenchymal, perivascular, and peribronchial regions of the lung. In regard to acute inflammation scores, which are characterized by the infiltration of neutrophils, only IN DTaP-vaccinated mice had a statistically significant reduction when compared with MVC mice (Fig. 4A). Interestingly, chronic inflammation scores, characterized by the infiltration of lymphocytes and plasma cells, were not significantly changed by either vaccine route or formulation (Fig. 4B). When compared with MVC mice, again, only IN DTaP mice showed a statistically significant reduction in total inflammation scores, which considers both acute and chronic scores together (Fig. 4C). In all categories of inflammation, the IN DTaP + BECC438b formulation incurred significantly higher inflammation scores than IN DTaP alone (acute *P* = 0.0476, chronic *P* = 0.0238, and total *P* = 0.0159). These data suggest higher recruitment of neutrophils, lymphocyte, and plasma cells to the lung tissue of mice vaccinated with BECC438b-adjuvanted formulations after *B. pertussis* challenge, which is consistent with a Th1-driven immune response. It is important to note that while inflammation levels in the BECC438b-adjuvanted vaccine groups were more similar to MVC mice than mice that had been vaccinated IM, the groups adjuvanted with BECC438b also had the lowest bacterial burden in the lungs following *B. pertussis* challenge (Fig. 1C). Together, these data might suggest that vaccine formulas containing BECC438b prime the immune microenvironment of the respiratory tract to produce a strong inflammatory response to *B. pertussis* challenge which limits bacterial burden. These data warranted further analyses to characterize the lung environment following BECC438b administration more deeply, in order to understand the unique anti-pathogen responses of vaccinated hosts.

The addition of BECC438b to DTaP increases the number of circulating immune cells in the blood but does not significantly alter the inflammatory cytokine profile of the lungs.

**Fig 5:**
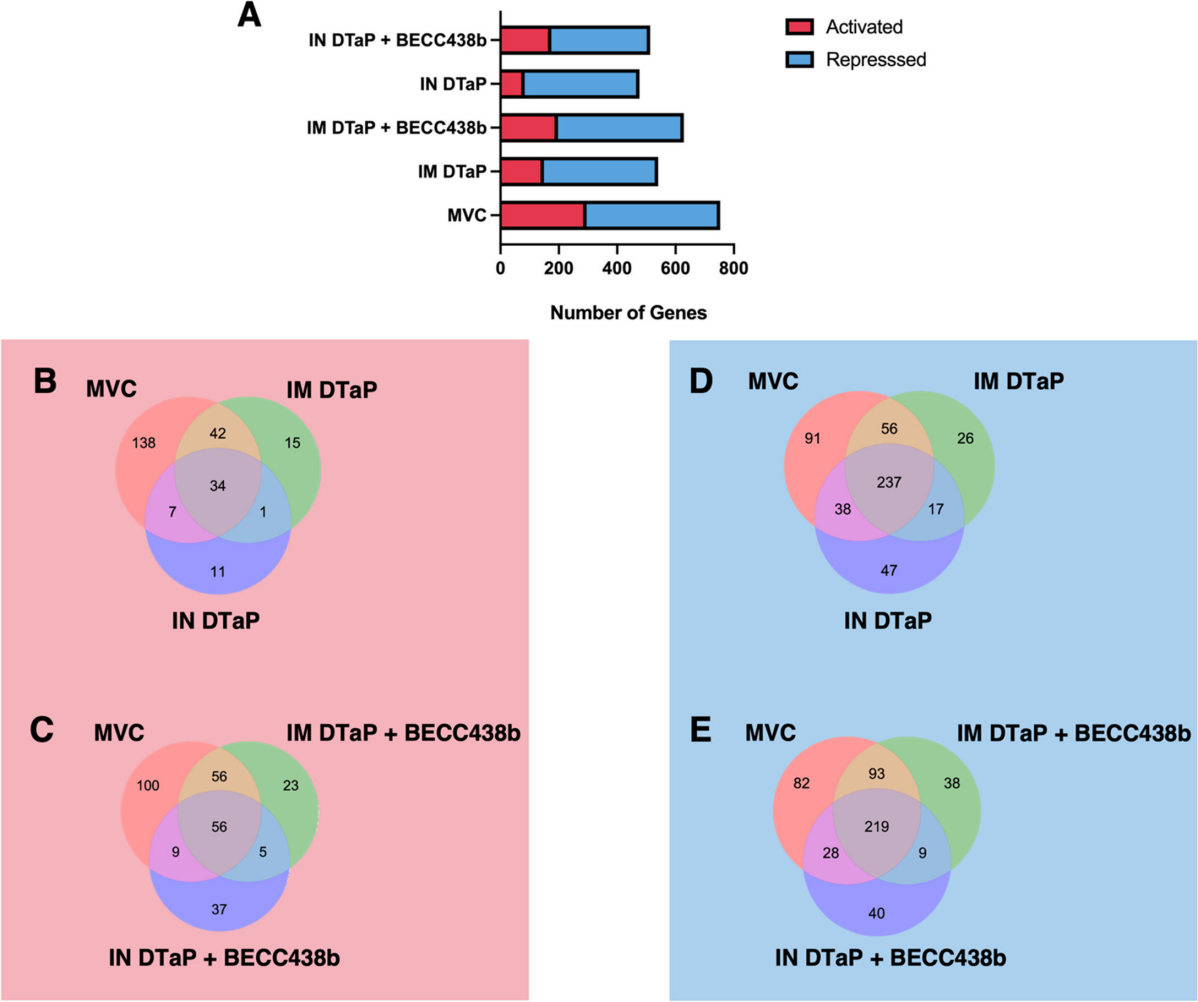
RNAseq analysis was performed to elucidate changes in genetic profiles between each experimental group. RNAseq analysis revealed the number of both activated (red) and repressed (blue) genes per experimental group. A bar chart representation of activated and repressed genes in shown in panel A. Venn diagrams which illustrate common activated genes between MVC mice and those vaccinated with DTaP (**B**) and MVC mice and those vaccinated with DTaP + BECC438b. All activated and repressed genes were deemed to be statistically different from NVNC mice via the Bonferoni *P*-value (*P* < 0.05). MVC, mock-vaccinated, challenged; NVNC, non-vaccinated, not-challenged; IM, intramuscular; IN, intranasal.

To further characterize the impact that administration of the BECC438b adjuvant with DTaP has on cellular responses, we performed a complete blood count with differential on blood collected from vaccinated mice at 3 days post-challenge. It is appreciated that the release of PT results in many of the symptoms associated with a pertussis infection, including leukocytosis (83). Like histological analysis (Fig. 4), we observed a statistically significant increase in both white blood cells and lymphocytes when the BECC438b adjuvant was added to DTaP formulations compared with what was seen from DTaP vaccination alone (Fig. S4A and B). Quantification of neutrophils within the blood revealed that all vaccination schemes resulted in statistically significant decreases in infiltration when compared with MVC mice (Fig. S4C). Monocyte quantification revealed no significant differences across both the control and vaccine groups (Fig. S4D). Furthermore, enumeration of cytokines within the pulmonary supernatant revealed that, when compared with MVC mice, all vaccination groups had a significant reduction in a number of cytokines typically associated with inflammation, such as TNF-α, IL-6, and IFN-γ (Fig. 5). BECC438b added to IM and IN DTaP did significantly increase CXCL13 levels compared with administration of DTaP by each route alone (IM vs IM *P* = 0.0372, IN vs IN *P* = 0.0073) (Fig. S5). Overall, the addition of BECC438b stimulates a strong initial immune response, as evidenced by the increased number of circulating immune cells; however, our data suggest that the production of proinflammatory cytokines within the lung remains similar among all vaccines, regardless of the presence of the BECC438b adjuvant.

### The addition of BECC438b and route of administration affects differential gene expression responses to DTaP

Serological responses to *B. pertussis* challenge in mice vaccinated by the IM route benefited from the addition of BECC438b; however, in IN vaccinated groups, antibody levels against *B. pertussis* antigens were very similar (Fig. 2). IN DTAP + BECC438b-vaccinated mice, however, were observed to have the lowest bacterial burden after challenge, with unique cellular inflammatory profiles in the lung (Fig. 4; Fig. S5). We hypothesized that administration of the combination vaccine formula to the nasal mucosa could be driving a different immune response to challenge in the mucosal tissues of the lung. In order to characterize the unique responses of each vaccinated cohort to challenge, we utilized total bulk RNAseq analysis to reveal the transcriptomic profiles. RNA reads were mapped to the mouse genome, and statistical analysis was performed to identify differentially expressed genes. We compared all gene expression profiles with NVNC control mice. Challenge with *B. pertussis* only (MVC mice) resulted in 295 genes activated and 458 genes repressed in the lung (Fig. 5A). On a surface level, regardless of the route of vaccine administration, the BECC438b adjuvant was able to increase the number of genes that were activated in the lung when compared with vaccination with DTaP alone (Fig. 5A). While IM vaccination with DTaP resulted in the activation of 149 genes, the addition of BECC438b to the formulation increased the number of activated genes to 197 genes (Fig. 5A). Similarly, IN DTaP alone activated 83 genes, while IN DTaP + BECC438b activated 174 genes (Fig. 5A). To begin to understand the effect that administration route plays in the unique gene expression profiles of BECC438b-vaccinated mice, we compared activated and repressed genes between groups. IN DTaP-vaccinated mice after challenge shared fewer activated genes in common with MVC mice than IM DTaP mice (7 genes shared vs 42 genes shared) (Fig. 5B). The addition of BECC438b mimicked this trend for IN and IM formulations, although the number of shared genes between each group increased slightly (Fig. 5C). IN DTaP + BECC438b vaccination resulted in more activated genes unique to the group post-challenge when compared with IM DTaP + BECC428b (37 compared with 23) (Fig. 5C). In repressed gene signatures, IN administration of DTaP had more unique repressed genes than IM DTaP after challenge, at 47 and 26 genes, respectively (Fig. 5D). In BECC438b formulations, less difference in the number of unique repressed genes was observed, with 40 unique to IN administration and 38 unique to IM (Fig. 5E). Despite these subtle differences, the overlap with genes activated or repressed in MVC mice is important to consider as well. As immunity can be gained from vaccination but also convalescence, similarities in transcriptomic responses to pathogen challenge may be useful for identifying the gene pathways that predict protection. IM vaccination, regardless of formulation with BECC438b, shared the highest number of activated and repressed genes with MVC, suggesting high similarity between the transcriptomic responses of IM vaccinated and unprotected hosts (Fig. 5). The least amount of similarity was seen between the expression profiles of IM and IN groups or IN and MVC groups, suggesting that route does dictate unique response profiles upon challenge (Fig. 5). These expression data begin to show again that IN DTaP and IN DTaP + BECC438b confer protection in response to *B. pertussis* challenge through unique pathways.

### The co-administration of BECC438b adjuvant alters downstream biological processes from *B. pertussis* challenge in vaccinated mice

**Fig 6:**
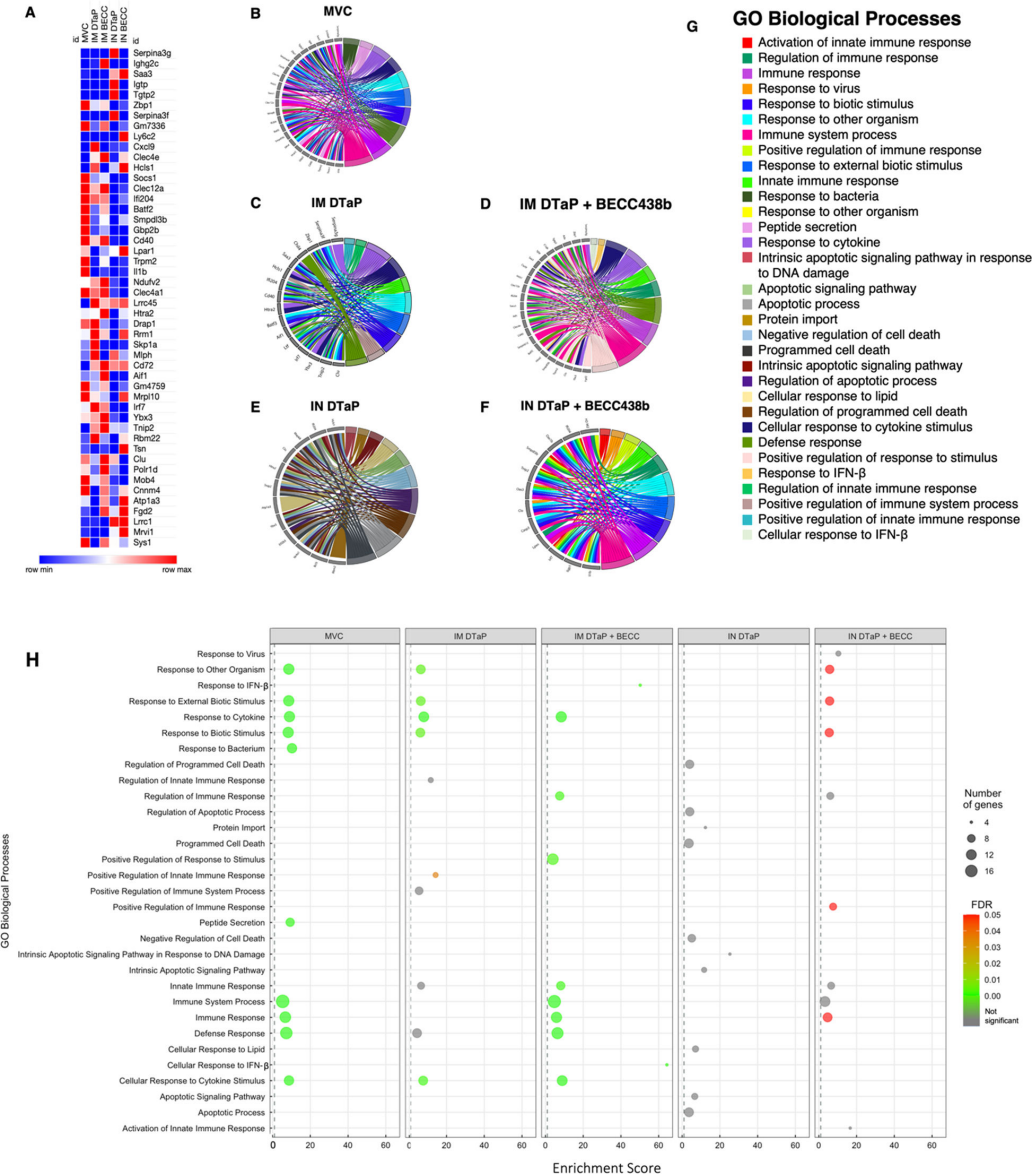
Transcriptomic and system analysis of mouse lungs at day 3 post *B. pertussis* challenge. Data from RNAseq analysis was used to determine the most common activated genes among all experimental groups and the downstream GO biological processes activated by those genes. Panel A depicts the top 40 activated genes shared by all experimental groups with blue boxes corresponding to minimum values, while red boxes correspond to maximum values. GO chord diagrams illustrated the GO biological processes activated by top activated genes were created for MVC- (**B**), IM DTaP- (**C**), IM DTaP + BECC438b- (**D**), IN DTaP- (**E**), and IN DTaP + BECC438b- (**F**) vaccinated mice. A key showing in which colors correspond to each GO biological process is shown in panel G. A bubble plot was created to show the biological processes activated by MVC mice as well as each vaccine group (**H**). The bubble plot shows the enrichment score, number of genes, and the FDR *P*-value of each pathway. Activated genes were determined to be statistically different from NVNC mice per the Bonferoni *P*-value (*P* < 0.05). The online tool, WEB-based GEne SeT AnaLysis, was used to determine the downstream biological processes associated with each activated gene.

After profiling the unique expression patterns of genes in the lung of vaccinated mice after *B. pertussis* challenge, we sought to evaluate the functions of activated and repressed genes. GO term analysis was used to determine the biological processes associated with the top 40 most highly activated genes in each of the vaccine route and formula groups (Fig. 6). The top 40 genes were then used to construct chord diagrams in order to determine changes in biological pathway activation between groups. As expected, MVC mice showed induction of many pathways that correspond to responses to a pathogen as well as immune system activation (Fig. 6B). DTaP administered IM resulted in the activation of pathways similar to that of MVC mice (Fig. 6C). The addition of BECC438b to the IM vaccine resulted in an increased number of immune system-involved pathways when compared with either MVC mice or mice vaccinated only with IM DTaP (Fig. 6D). Evaluation of IN administration of DTaP showed a marked activation of pathways involved in apoptosis or the regulation of apoptosis (Fig. 6E). However, it is of note that while the genes that were activated were all significantly different from NVNC per Bonferroni analysis, none of the GO biological pathways were considered statistically significant for IN DTaP-vaccinated mice, as determined by the FDR *P*-value (Fig. 6H). With mice vaccinated with IN DTaP + BECC438b, there was a shift back to the activation of pathways that tied into the immune system, whether that be via activation of regulation (Fig. 6F). Additionally, unlike IN DTaP vaccination, the addition of BECC438b resulted in the activation of pathways that were considered significant per the FDR *P*-value (Fig. 6H). Overall, these data support that the IN vaccination route resulted in unique pathway activation in response to pathogen challenge and support our earlier observations that BECC438b is able to increase immune system activation when added to the base DTaP vaccine, regardless of the route of vaccine administration.

### Intranasal administration of DTaP + BECC438b increases the total counts of immunoglobulin genes in the lung

**Fig 7:**
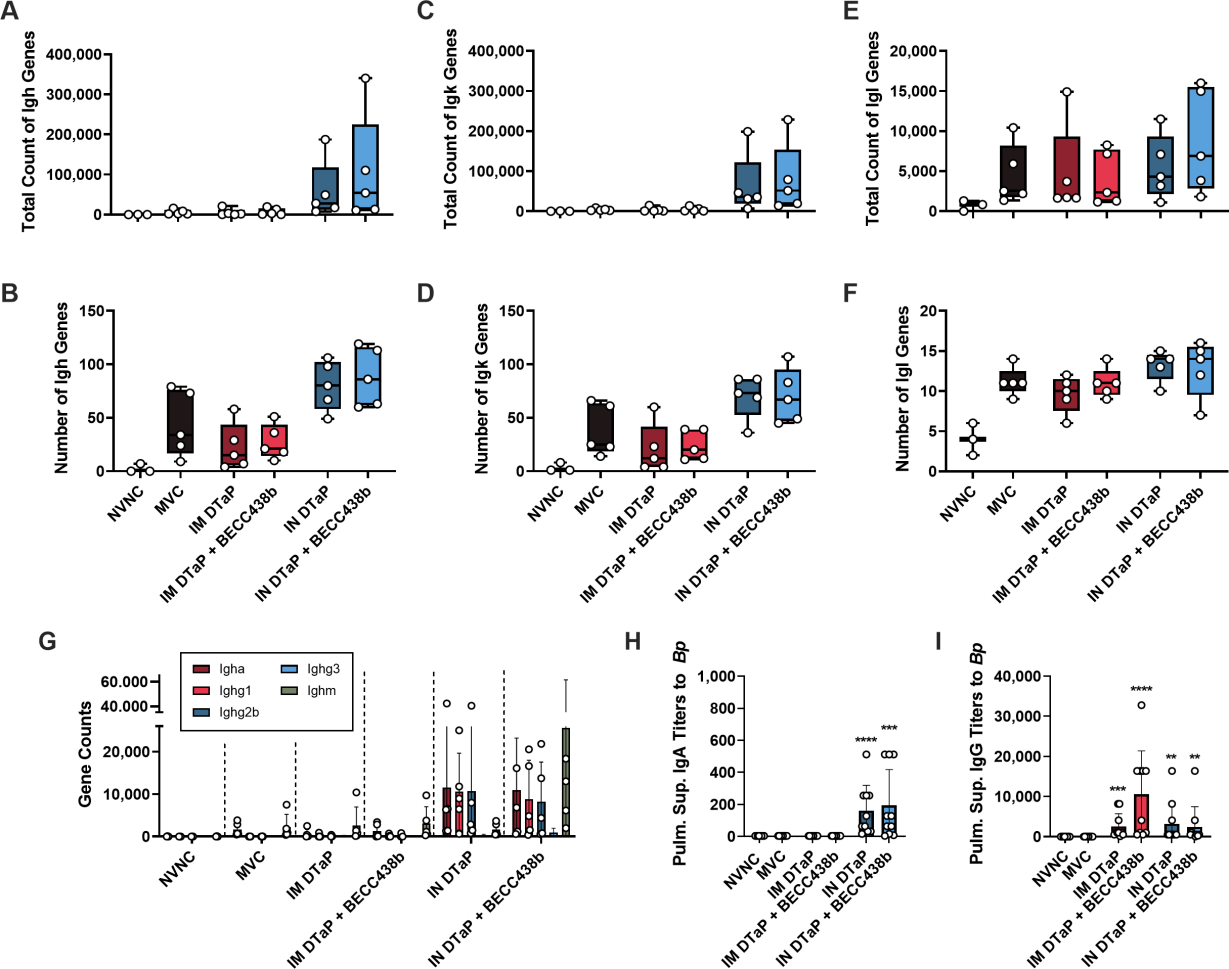
Analysis of antibody genes expressed in mouse lungs at day 3 post *B. pertussis* challenge. Total gene counts were determined for *Igh* (**A**), *Igk* (**B**), and *Igl* (**C**) genes. Additionally, we looked at the number of unique genes within the lung tissue for *Igh* (**D**), *Igk* (**E**), and *Igl* (**F**) immunoglobulin genes. To evaluate the number of constant genes within each vaccine groups, we evaluated the number of gene counts for each heavy chain (**G**). To corroborate the heavy-chain gene count data, we performed ELISA assays to enumerate the amount of IgA antibody within the pulmonary supernatant (**H**) as well as the amount of IgG antibodies within the pulmonary supernatant (**I**). The data represent the total number of genes for all heavy- and light-chain antibodies, including all variants. Data presented as geometric means ± SD, *n* = 5 per treatment group. The exception to this is ELISA data, in which *n* = 10 per treatment group. Kruskal-Wallis tests were performed to determine statistically significant differences in MVC mice and all other treatment group. **P < 0.0071, ***P = 0.0003, and ****P < 0.00011. Mann-Whitney tests were used to determine statistically significant differences between each vaccine route and formulation. MVC, mock-vaccinated, challenged; NVNC, non-vaccinated, not-challenged; IM, intramuscular; IN, intranasal.

The RNAseq data illustrated the base ability of IN vaccination of DTaP alone or to a higher degree with the inclusion of the BECC438b adjuvant to activate pathways involved in the immune system such as the innate immune response, regulation of immune system processes, and the overall regulation of the immune response (Fig. 6). As previously stated, antibodies are a critical player in vaccine-elicited protection against *B. pertussis* exposure or challenge. To more closely profile the effect that these vaccines had on responses to *B. pertussis* challenge in our mouse model, we referred to our complete gene expression browser to examine the genes required for immunoglobulin production after vaccination. There are two ways that immunoglobulin genes could change in expression when evaluating an organ’s overall RNA transcriptome: (ii) cells are recruited that express immunoglobulin genes or (ii) immunoglobulin genes of existing cells are increased. We hypothesized that the detection of immunoglobulin genes in the lung tissue reflects the presence of B cells, potentially plasmablasts or plasma cells. Specifically, we wanted to determine changes within the B cell receptors, which is comprised of a heavy chain (Igh) and one of two light chains (Igk or Igl). RNA reads corresponding to *Igh*, *Igk*, and *Igl* variable genes were analyzed to determine the breadth of immunoglobulin genes being expressed. In the total counts of Ig genes, we observed a marked increase in the number of both *Igh* and *Igk* genes from IN vaccinated mice with IN DTaP + BECC438b having the highest counts overall (Fig. 7A and B). These counts corresponded to an increase in the number of *Igh* genes as well, suggesting a greater diversity of gene activation (Fig. 7C and D). Interestingly, while the counts of *Igh* and *Igk* genes were negligible in the IM vaccination groups, the total count of *Igl* genes in IM vaccinated mouse lungs increased to a level comparable to both IN vaccination groups (Fig. 7E). Similar diversity of *Igl* genes was observed across vaccine groups as well (Fig. 7F). Of the genes with the highest overall counts, a vast majority were immunoglobulin constant genes; therefore, we enumerated the number of gene counts of each Ig constant within each vaccine group. As seen in previously discussed data, immunoglobulin constant gene counts were almost negligible in the lungs of *B. pertussis*-challenged mice unless they had been previously vaccinated by the IN route (Fig. 7G). Within the IN DTaP only group, the gene counts were highest for *Igha*, *Ighg1*, and *Ighg2b*, while, in mice vaccinated IN with DTaP adjuvanted with BECC438b, there was a large increase in the number of *Ighm* genes (Fig. 7G). To further corroborate these findings, we performed ELISAs on the pulmonary supernatant to quantify the level of IgA and IgG within these respiratory tissues. Challenge with *B. pertussis* did not induce IgA production in the lung of nonvaccinated mice (Fig. 7H; Fig. S7). In line with the observation of increased gene counts, there was an increase in IgA in mice that received IN vaccination, both DTaP formulations alone and including BECC438b (Fig. 7H). IN DTaP + BECC438b also induced an increase in IgA detectable in the nasal wash of mice (*P* < 0.0001 compared with NVC); however, this was at a very low titer. IgG titers in the lung were insignificantly affected by prior IN vaccination, regardless of formulation (Fig. 7I). Interestingly, BECC438b did demonstrate its potential effects on lung IgG antibody production, as IM DTaP + BECC438b mice had the most robust IgG antibody level when compared with all other vaccine groups (Fig. 7I). It is interesting to note that we did not detect significant counts of IgG genes (*Ighg1*, *Ighg2b*, and *Ighg3*) (Fig. 7G) despite detecting IgG antibodies in the serum (Fig. 2) and pulmonary supernatant after challenge (Fig. 7I). Surprisingly, quantitation of IgM antibodies in the pulmonary supernatant showed that the presence of anti-*Bp* IgM was negligible across all experimental groups, which is in contrast to what was expected given the high number of *Ighm* reads seen in mice vaccinated IN with DTaP + BECC438b (data not shown). Overall, we were able to more deeply examine the serological data from this study and highlighted distinct antibody responses that were unique to each vaccine formulation and route. Specifically, it was observed that induction of mucosal immune responses, measured by the production of IgA and IgG antibodies in the lung after *B. pertussis* challenge, is largely dependent upon prior IN DTaP immunization and can be enhanced by the BECC438b adjuvant. The presence of *B. pertussis*-specific IgA and gene reads for IgA constant suggests that IN immunization with and without BECC438a can result in antibody production in the lungs which can theoretically be protective during respiratory pathogen challenge. Despite these similarities in antibody data from IN vaccinations, IN DTaP and IN DTaP + BECC438b still differed in their ability to control bacterial burdens in the tissues post-challenge (Fig. 1). Further characterization of this antibody producing immune cell population would be necessary to ascertain if these cells are tissue resident and produced due to immunization or if they were recruited to these tissues due to challenge.

## DISCUSSION

In this study, we sought to evaluate two different approaches for improving the protection of aP vaccines: (i) administration of vaccine by the IN route to activate mucosal immunity and (ii) co-formulation with the BECC438b TLR4 agonist adjuvant to sway DTaP-induced responses from Th2 phenotypes toward Th1. Female CD-1 mice were primed and boosted with either 1/40th the human dose of DTaP alone or 1/40th DTaP + BECC438b. IN administration was compared with IM administration, to represent the standard vaccination method used for humans. Mice were then challenged with *B. pertussis* and processed at 3 days post-challenge. We first observed that IN administration of DTaP alone reduced the bacterial burden in the nasal lavage fluid and lung tissue of vaccinated mice subjected to intranasal challenge with a liquid inoculum of *B. pertussis* (Fig. 1). The addition of BECC438b to DTaP in IN-administered formulations did not significantly reduce bacterial burdens further in the nasal cavity (determined via analysis of the NALT and nasal lavage fluid), despite our hypothesis that changing the route and formulation together would confer the greatest protection. However, we did observe that IM and IN formulations containing BECC438b conferred the highest protection against lung colonization in our mouse model, indicative of protection against more severe disease (Fig. 1). Together, these observations of bacterial burden throughout the respiratory tract showed that DTAP administered by the IN route can limit *B. pertussis* burden as well as IM administration. The addition of the BECC438b adjuvant also showed its utility in improving protection in a tissue-dependent manner.

We continued to observe subtle differences between vaccine routes and formulations in the analyses of humoral vaccine responses. Both IN and IM administration of DTaP elicited comparable levels of IgG antibodies specific to *B. pertussis* whole bacterium and FHA and PT antigens. BECC438b improved anti-*B. pertussis* IgG levels compared with DTaP alone when administered IM. IM DTaP + BECC438b mice also exhibited the highest titers against PRN across vaccine groups (Fig. 2). These data may help support the immunostimulatory potential of the BECC438b adjuvant, as PRN is present in DTaP at the lowest concentration of all included antigens.

Our studies aimed to use BECC438b to shift cellular immune responses against DTaP toward a Th1 phenotype which, in the case of wP pertussis vaccines, has been determined to confer desirable protection. During pertussis infections, it has also been established that cell-mediated immunity plays a large role in the clearance of *B. pertussis*, highlighting a correlate of protection which vaccines should meet (7, 41). When IgG isotype analysis was performed on pre-challenge sera, it was observed that BECC438b had no effect on changing the immune response to IM-administered DTaP (Fig. 3). It was only when BECC438b was administered with DTaP IN that immune responses shifted, eliciting the more desirable Th-1 polarized response, indicated by an increase in the IgG2b antibody subtype (Fig. 3). It is interesting that this effect could only be seen after IN BECC438b vaccination. This could be an effect of the delivery pathway, where the vaccine activates the inflammatory pathways of the mucosa in a manner replicating how *B. pertussis* would during natural infection. IN DTaP alone may benefit from the proinflammatory effects of BECC438b, which can in theory trigger TLR4-induced production of IFN-γ, a Th1 cytokine, from cells in the respiratory mucosa and activate the immune cells which will process and present vaccine antigen for the development of memory responses (84). Furthermore, as this vaccine formulation contains both alum and BECC438b, it could be that the BECC438b adjuvant takes advantage of the suspected depot effect of alum, allowing the TLR4 agonist to remain localized at the site of vaccination in the nares for a prolonged period of time, supporting the mucosal immune response. (85) This prolonged period of inflammation with antigen processing may be supportive of the increase in IgG2b observed in our mouse cohorts; however, analysis of the cellular effects of BECC438b on the respiratory tract is outside the scope of this study’s data.

In an attempt to understand the more complex, yet subtle, effects of BECC438b co-formulation on the immune response to DTaP, we performed RNAseq analysis on lung tissue from vaccinated mice after *B. pertussis* challenge. The inclusion of BECC438b increased the number of activated genes when administered IM or IN. Within these groups, gene expression profiles were more similar between IM vaccinated groups and MVC mice than IN and MVC groups with more shared genes activated or repressed (Fig. 5). While the IM DTaP-shaped transcriptome suggested biological pathway activation related to robust responses to other organisms, stimuli, and cytokines, the inclusion of BECC438b shifted the response to one that had major involvement of immune system-related pathways as well (Fig. 6). This was expected, as the TLR4 pathway activates not only the release of pro-inflammatory cytokines but also the activation of both innate and adaptive immune cells (86). Additionally, while IN administration of DTaP had no pathways activated that were deemed to be statistically significant, the addition of BECC438b to IN DTaP significantly increased the activation of biological pathways associated with responses to stimuli and immune system activation. To further corroborate this transcriptomic data, future experiments could consider confirming gene expression profiles via qRT-PCR analysis, choosing target genes related to the desirable inflammatory responses BECC438b is hypothesized to affect.

While the antibody titer data did illustrate variable increases in serum antibodies upon the use of BECC438b, the RNAseq gene count data were able to illustrate even more differences between vaccine groups. Rather than focusing on the variability that is associated with light-chain genes, we instead wanted to focus on the antibody heavy chains or the constant regions. Interestingly, the highest counts associated with heavy chains were seen in the lungs of IN vaccinated mice. Both IN vaccination groups had elevated counts of *Igha*, *Ighg1*, and *Ighg2b* (Fig. 7). The increased level of *Igha* we observed is expected and a highly desirable result from mucosal vaccination. The *Ighg1* and *Ighg2b* results are indicative of a mixture of Th1- and Th2-polarized immune response, which is expected to occur from both the aP vaccine formulation and BECC438b. Of interest is the ability of IN DTaP + BECC438b to cause such a larger increase in *Ighm* gene counts. IgM is the first antibody to respond to an encounter with a foreign pathogen and is an activator of the complement cascade (87). Additionally, IgM contains a J-chain, which allows the antibody to interact with pIgR, meaning it can access the mucosal lumen (88). Previous studies have suggested that immunity within the lung is dependent upon a subset of B cells known as B-1 cells, which secrete large amounts of secretory IgM (89). With this knowledge, the increased gene counts of *Ighm* are interesting to see as a result of IN vaccination with DTaP + BECC438b, as it may be indicative of a more robust mucosal immune response expanding the antibodies induced by vaccination to include more than just IgA antibody. However, though we observed high gene counts, ELISA assays to corroborate this data did not show appreciable levels of IgM antibody in the pulmonary supernatant (data not shown). While we looked for antibodies that were specific to the whole pertussis bacterium in ELISAs, it could be that the high counts of *Ighm* and other genes could be antibodies specific to other antigens included in DTaP such as diphtheria toxoid or tetanus toxoid. Additional assays would need to be performed to identify and classify these antibodies.

Based on our data presented here, we hypothesize that intranasal administration of DTaP with BECC438b potentially induces memory lymphocyte clusters in the mucosa that can participate in bacterial detection and clearance. RNAseq analysis of gene counts within each vaccination group revealed a marked increase in *Cd69* gene expression in mice IN vaccinated with DTaP + BECC438b (426-fold activated over NVNC; *P* = 0.002). CD69 is expressed on cells that are classified as “tissue-resident cells,” and it is considered to be the marker of T resident memory cells (T_RM_s) (90). T_RM_s remain localized at a particular location (such as the lung, the skin, or genital mucosa) and serve as the first line of defense against a pathogen (91). Previous studies support that the clustering of these T_RM_s along with antigen-presenting cells may play a role in protecting against subsequent infections with a pathogen (92, 93). We speculate that IN administration of the BECC438b adjuvant supported the development of memory lymphocyte clusters in the lung tissue, which explains why these mice, when compared with all other vaccine groups, had greater induction of downstream immune response pathways and such a striking number of *Ighm* reads in the lung after challenge (Fig. 7). One confounding factor to consider is that the immunoglobulin gene expression data obtained from RNAseq is not specific for *B. pertussis* antigens. These antibody genes could possibly suggest that additional immune pathways are being activated and that B cells are existing in the lungs after challenge. It is possible that nonspecific immune activation is occurring simultaneously, as was seen with BPZE1, an intranasal pertussis vaccine candidate found to be cross-protective against lethal challenge with *Streptococcus pneumoniae* (94). To determine the contribution of T cells in the lung to the unique immune responses observed in BECC438b-vaccinated mice, additional studies should be performed utilizing flow cytometry to identify T_RM_s post-vaccination preluding challenge in addition to after challenge with *B. pertussis.*

Future studies to more clearly define the immunostimulatory effects of BECC438b during vaccination against respiratory pathogens are warranted to better understand the data observed in this study. BECC technology used as adjuvants in vaccines against respiratory diseases like influenza has been explored previously with great success (95, 96). While these applications did not explore route of administration, we believe our data prepare a case for its efficacy in novel intranasal vaccines. To enable these applications, future study design should consider a number of variables absent here. Our challenge model of *B. pertussis* results in a large deposit of bacteria into the nasal cavity—much more than what would be seen with natural infection. Despite presenting a more realistic exposure pathway, bacterial burdens from aerosol challenge appeared similarly high in each tissue. While all of the tested vaccines resulted in some level of protection, indicated by a lower number of bacteria within the respiratory tract when compared with MVC mice, the variation between biological replicates within groups for some organs clouded positive trends in the data. In one strange observation of bacterial burden in the nasal cavity, the addition of BECC438b to the vaccine formulation appeared to result in an increase in bacterial burden (Fig. S2). This was also the case for IN immunization and bacterial burden in the lung and trachea (Fig. S2). Current *B. pertussis* vaccines carry room for improvement in their ability to prevent bacterial carriage in the upper respiratory tract which would prevent transmission. Despite detecting bacterial loads in the upper respiratory tract of mice, we are unable to assess transmission from vaccinated carrier hosts in mice that do not transmit respiratory pathogens between cage mates. Studies utilizing transmission models like the Syrian hamster may be useful to analyze this variable with different doses of vaccine and adjuvant: while we believed that a biologically appropriate dose of DTaP for mice is 1/40th human dose, it may confer too low a level of protection for our challenge dose or may possibly be too high to be able to measure the added benefit from adjuvants. In another study by our lab which investigated the longevity of protection afforded by aP vaccines in the mouse model, 1/20th human dose was used as the base for formulations incorporating adjuvants (30). This may be appropriate to employ here for BECC438b as well. It is also quite possible that our selected dose of BECC438b was low and that a greater effect could be measured at higher concentrations.

An important consideration for any preclinical work in mice is that mice and humans vary greatly in the anatomy and structural architecture of their respiratory tracts, as well as in the timing of their immune responses. This provides a caveat for challenge models, which largely may impact the clearance that we can observe from vaccination, regardless of the bacterial deposition effect from conferring challenge (59). Also, we only utilized one time point post challenge: 3 days. Our lab has previously showed that the 3-day-post-challenge timepoint regardless of aerosolization or intranasal instillation corresponds to a peak in bacterial burden in disease-relevant tissues (66, 67). This does not necessarily correlate to peaks in antibody or cellular responses which may grow with time. In humans, antibody levels against pertussis vaccine antigens begin to wane each year after receiving a booster vaccination (97). In mice, however, the antibody levels within the sera are maintained and do not seem to wane long after vaccination and our previous studies have utilized this particular data collection point (28, 30, 66). Furthermore, we know that beyond this time point, the bacterial burden begins to decrease, as adaptive responses take effect and clear the pathogen even without prior vaccination. Regardless, as a result of this study, we cannot attest to the long-term protection afforded by these formulations, but by studying this time point, we are able to understand the immediate effects of existing vaccine immunity on challenge.

This work explores two major considerations for the improvement of aP vaccines which are accepted as safer and less reactogenic than wP vaccines, at the cost of conferring reduced levels of immunity. We observed in a murine model of vaccination and challenge that changing the route of administration for the DTaP aP vaccine from intramuscular to intranasal may offer equal if not better levels of protection against bacterial burden in the tissues despite induction of a unique profile of humoral immune correlates. IN vaccination induced mucosal IgA responses, as expected, in addition to limited inflammation in the lung tissue after challenge which we believe is indicative of the successful activation of mucosal immunity. We also explored the utility of a novel adjuvant, BECC438b, in shifting aP-induced immunity to a Th1 phenotype which is more desirable for anti-*B. pertussis* immunity. IN vaccination with DTaP + BECC438b resulted in a Th1-polarized immune response, while IM vaccination with or without adjuvant maintained the Th2 response associated with aP vaccine formulations. Finally, IN vaccination with DTaP + BECC438b was able to change the downstream activation of biological pathways, shifting aP responses to become similar to what is seen with natural infection. While this is only the first step in evaluating BECC438b against *B. pertussis* challenge, it is beneficial to know that BECC438b likely works cohesively with alum, meaning that there is no need to deconstruct the existing formulation. Furthermore, as we continue to work to develop the next generation of pertussis vaccines, we will continue to evaluate IN immunization. As more studies are completed on BECC438b, in terms of immunogenicity, safety, and performance in non-human primate models, we remain hopeful that it could become a candidate for inclusion in future vaccines for pertussis or other diseases.

## Data Availability

Data for all figures are available upon reasonable request to the corresponding author. Fastq raw read data are deposited at NCBI SRA under Bioproject number PRJNA909838. The analyzed RNAseq data table in CLC file format and Excel file format is deposited at Mendeley Data (DOI: 10.17632/gdtx97tj3t.1).

## SUPPLEMENTAL MATERIAL

Fig. S1 iai.00223-23-s0001.tif Graphic of experimental design.

Fig. S2 iai.00223-23-s0002.tif Bacterial burden from aerosol challenge experiment.

Fig. S3 iai.00223-23-s0003.tif OD values from spectrophotometer readings to determine IgG antibody titers.

Fig. S4 iai.00223-23-s0004.tif Complete blood count 3 days post-challenge.

Fig. S5 iai.00223-23-s0005.tif Cytokines and chemokines in the lung 3 days post-challenge.

Fig. S6 iai.00223-23-s0006.tif OD values from spectrophotometer readings to determine IgG subclass antibody titers.

Fig. S7 iai.00223-23-s0007.tif OD values from spectrophotometer readings to determine IgA antibody titers.

ASM does not own the copyrights to Supplemental Material that may be linked to, or accessed through, an article. The authors have granted ASM a non-exclusive, world-wide license to publish the Supplemental Material files. Please contact the corresponding author directly for reuse.
