# Supplementary figures and images for: BECC438b TLR4 agonist supports unique immune response profiles from nasal and muscular DTaP pertussis vaccines in murine challenge models
Source: Infect Immun. 2024 Feb 7;92(3):e00223-23. doi: 10.1128/iai.00223-23 (PMC10929442; doi:10.1128/iai.00223-23)

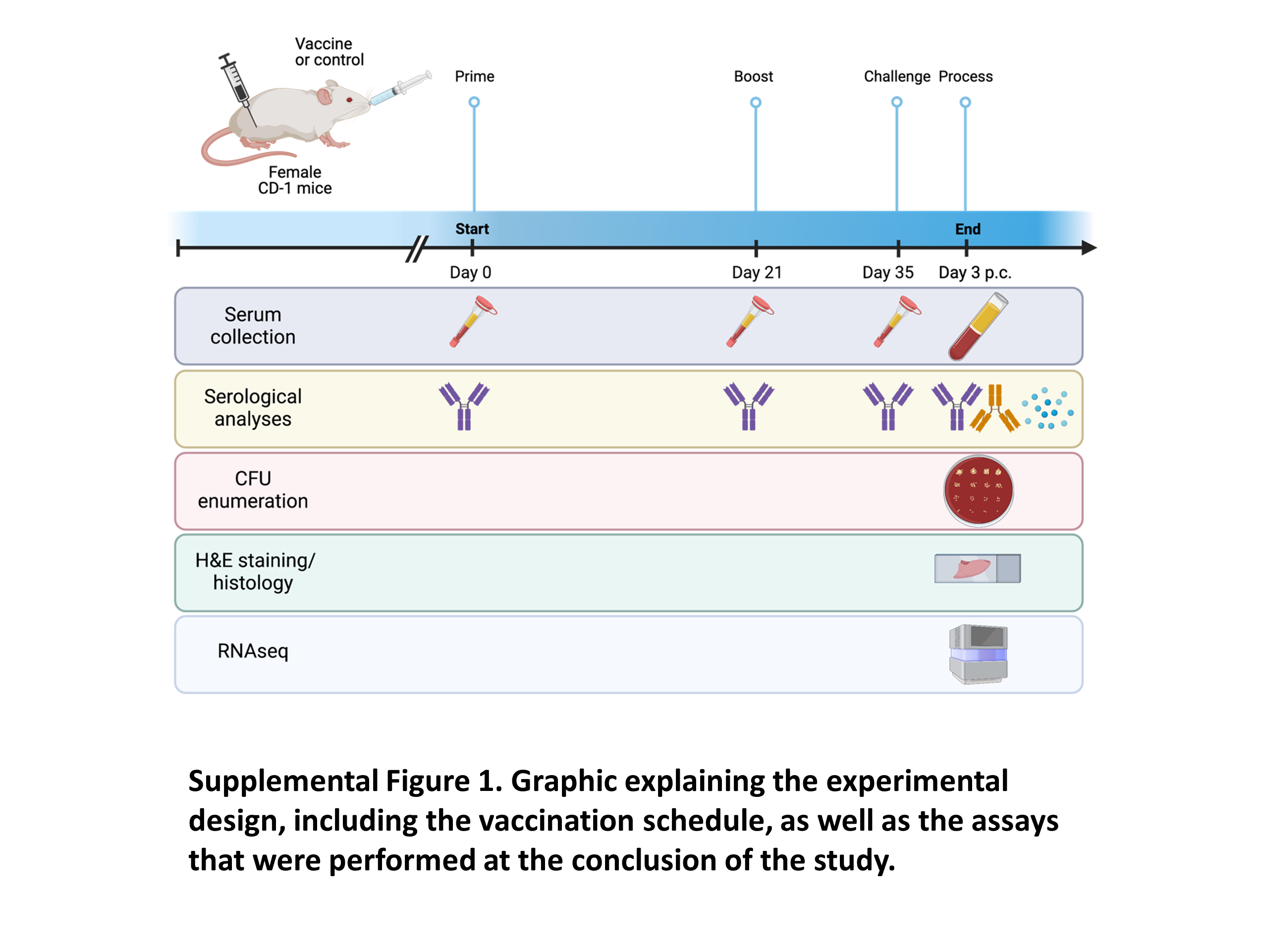

Supplement: Fig. S1 — Graphic of experimental design. [file iai.00223-23-s0001.tif]

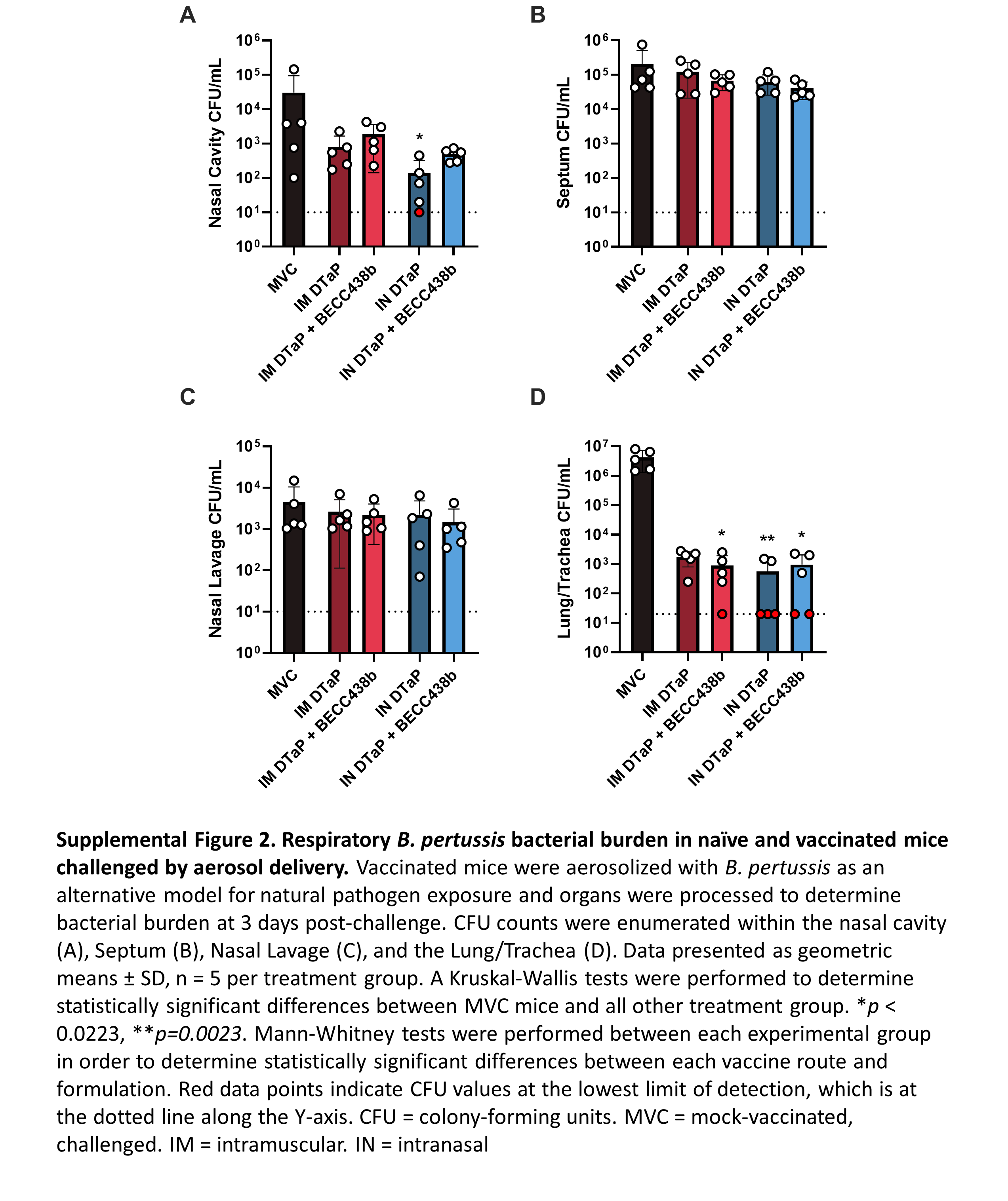

Supplement: Fig. S2 — Bacterial burden from aerosol challenge experiment. [file iai.00223-23-s0002.tif]

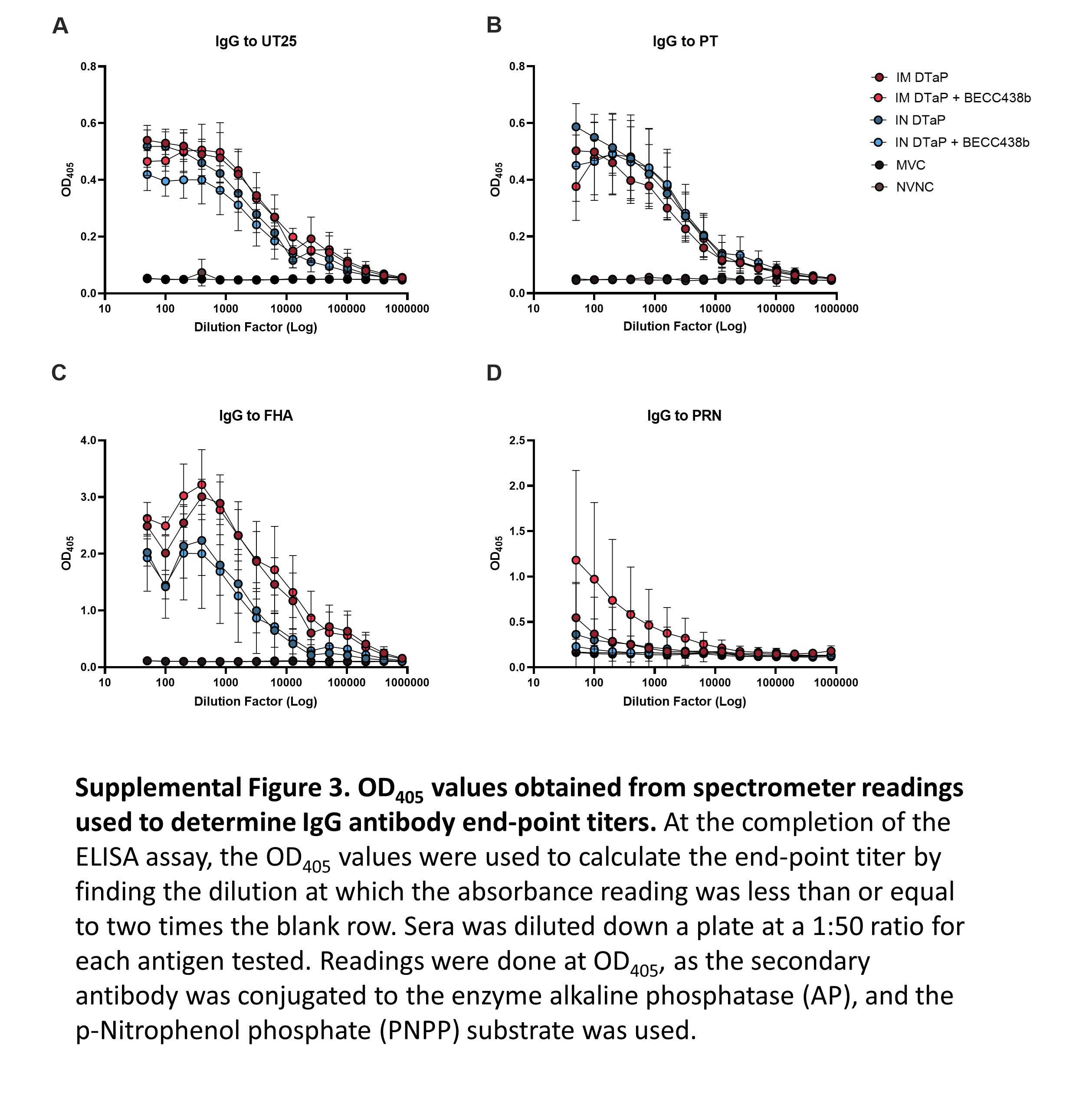

Supplement: Fig. S3 — OD values from spectrophotometer readings to determine IgG antibody titers. [file iai.00223-23-s0003.tif]

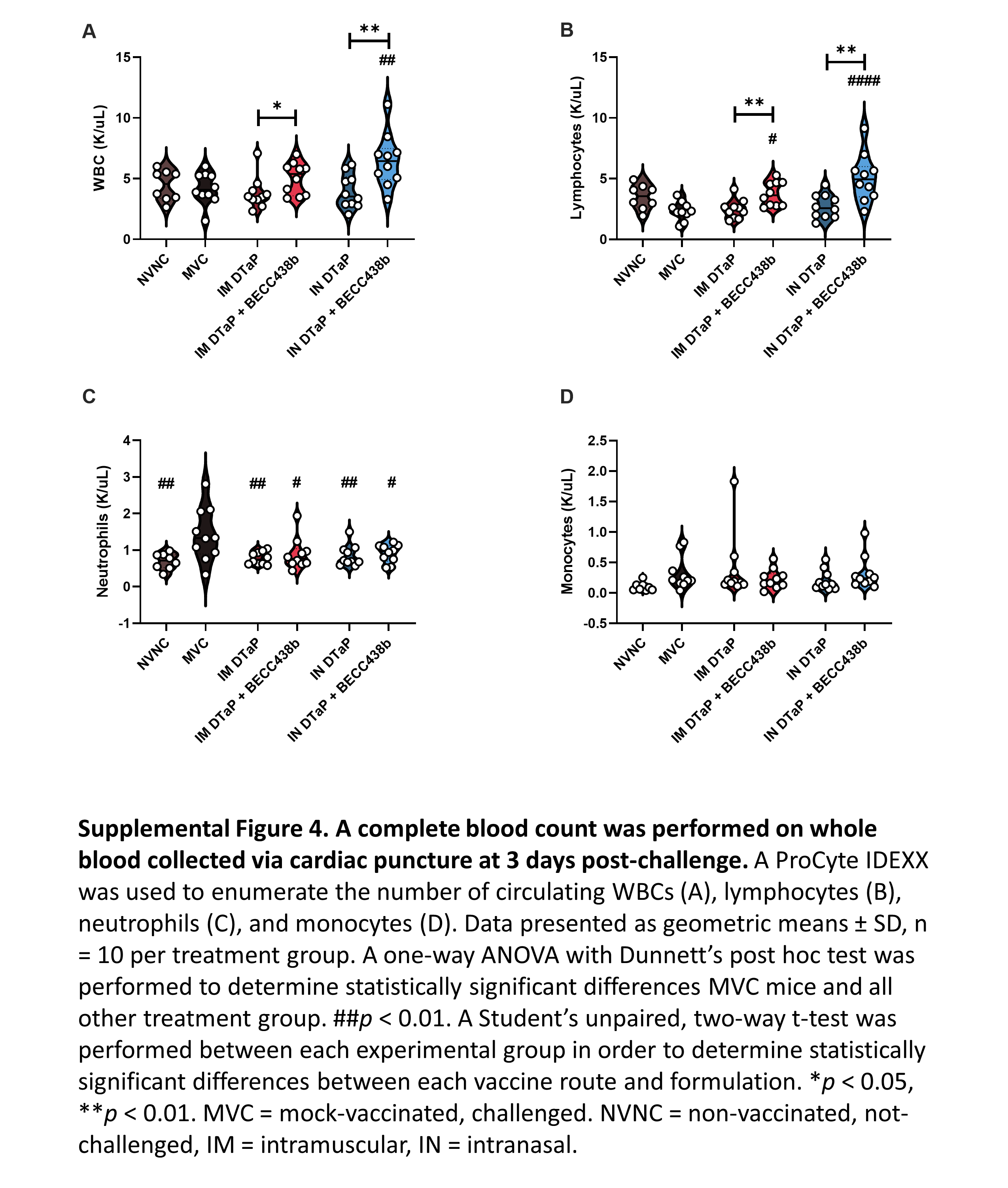

Supplement: Fig. S4 — Complete blood count 3 days post-challenge. [file iai.00223-23-s0004.tif]

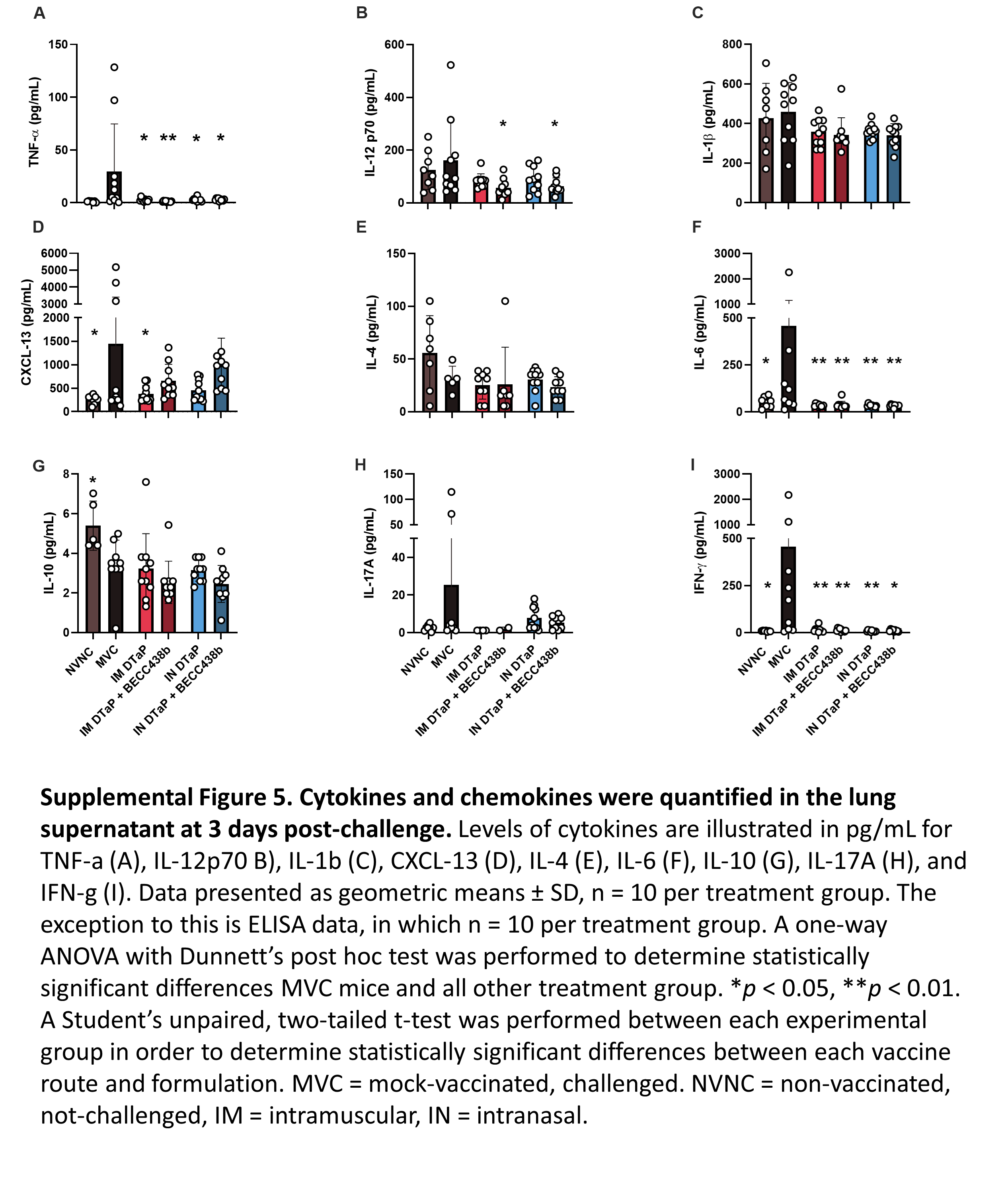

Supplement: Fig. S5 — Cytokines and chemokines in the lung 3 days post-challenge. [file iai.00223-23-s0005.tif]

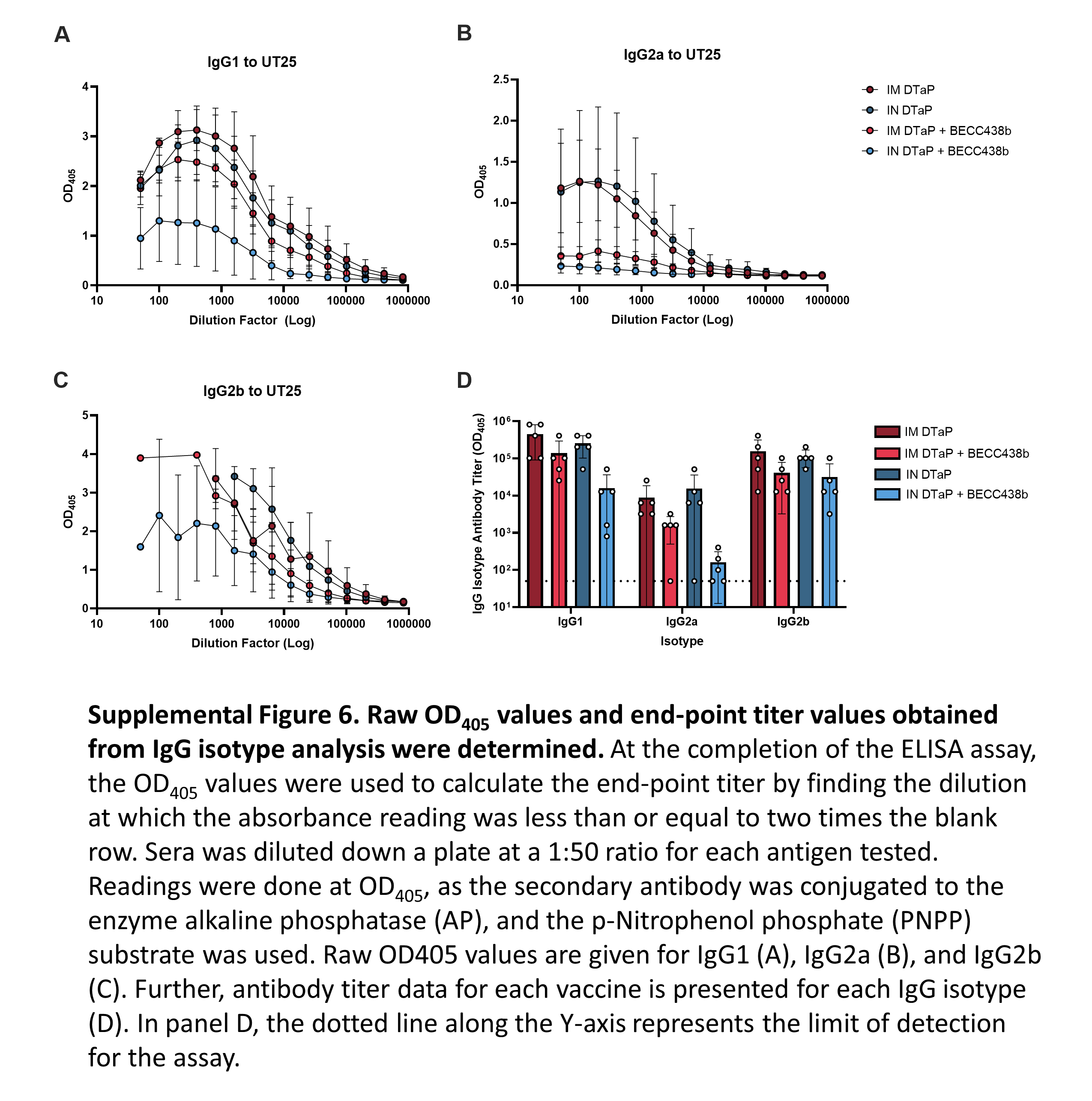

Supplement: Fig. S6 — OD values from spectrophotometer readings to determine IgG subclass antibody titers. [file iai.00223-23-s0006.tif]

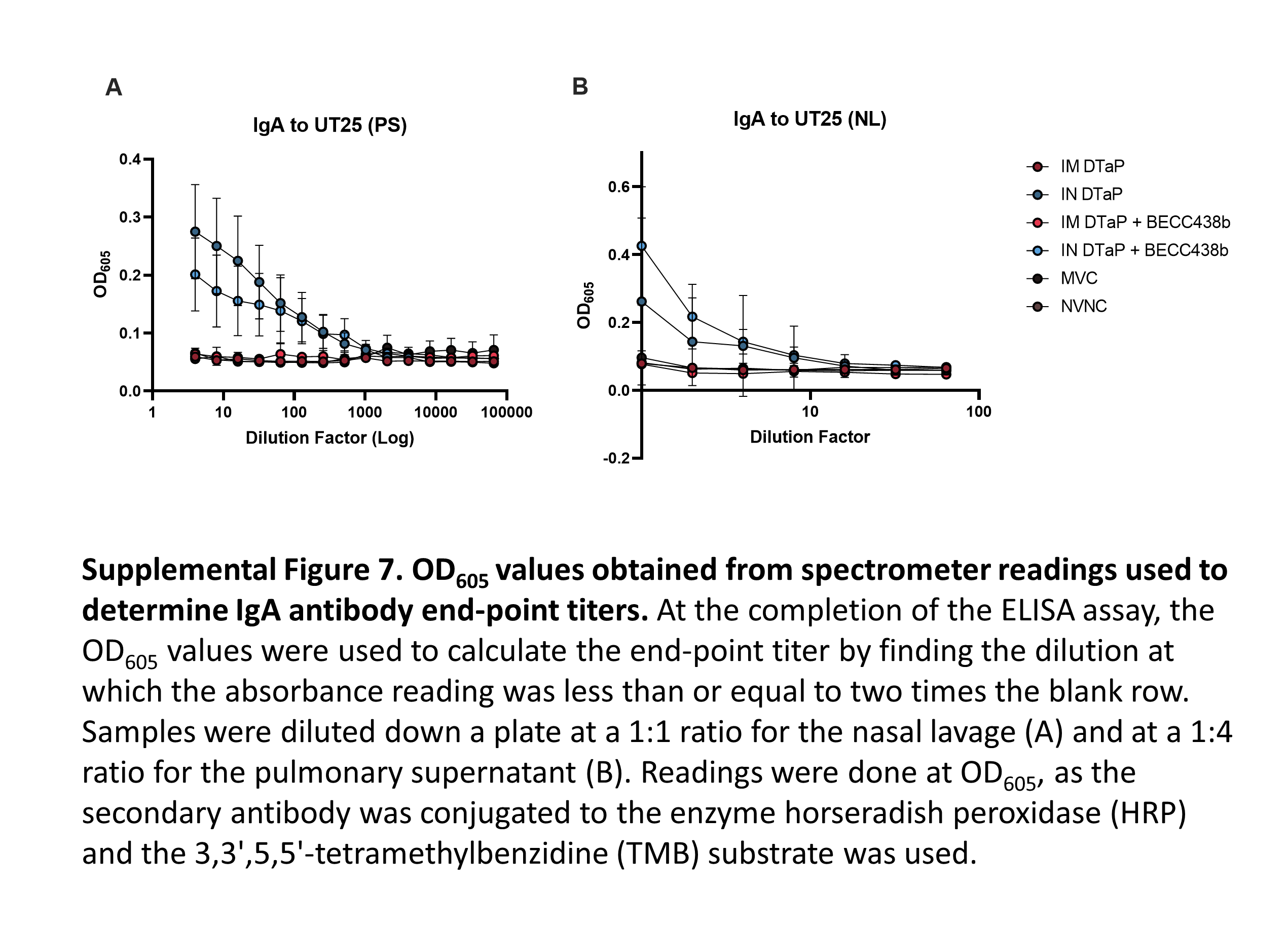

Supplement: Fig. S7 — OD values from spectrophotometer readings to determine IgA antibody titers. [file iai.00223-23-s0007.tif]
